# Genome-scale analysis of *Arabidopsis* splicing-related protein kinase families reveals roles in abiotic stress adaptation

**DOI:** 10.1186/s12870-022-03870-9

**Published:** 2022-10-22

**Authors:** M. C. Rodriguez Gallo, Q. Li, D. Mehta, R. G. Uhrig

**Affiliations:** 1 Department of Biological Sciences, University of Alberta, Edmonton, AB T6G 2E9 Canada; 2 Department of Biochemistry, University of Alberta, Edmonton, AB Canada

**Keywords:** Abiotic Stress, Evolution, Diel cycle, mRNA splicing, Protein kinases

## Abstract

Nearly 60 – 80 % of intron-containing plant genes undergo alternative splicing in response to either stress or plant developmental cues. RNA splicing is performed by a large ribonucleoprotein complex called the spliceosome in conjunction with associated subunits such as serine arginine (SR) proteins, all of which undergo extensive phosphorylation. In plants, there are three main protein kinase families suggested to phosphorylate core spliceosome subunits and related splicing factors based on orthology to human splicing-related kinases: the SERINE/ARGININE PROTEIN KINASES (SRPK), ARABIDOPSIS FUS3 COMPLEMENT (AFC), and Pre-mRNA PROCESSING FACTOR 4 (PRP4K) protein kinases. To better define the conservation and role(s) of these kinases in plants, we performed a genome-scale analysis of the three families across photosynthetic eukaryotes, followed by extensive transcriptomic and bioinformatic analysis of all *Arabidopsis thaliana* SRPK, AFC, and PRP4K protein kinases to elucidate their biological functions. Unexpectedly, this revealed the existence of SRPK and AFC phylogenetic groups with distinct promoter elements and patterns of transcriptional response to abiotic stress, while PRP4Ks possess no phylogenetic sub-divisions, suggestive of functional redundancy. We also reveal splicing-related kinase families are both diel and photoperiod regulated, implicating different orthologs as discrete time-of-day RNA splicing regulators. This foundational work establishes a number of new hypotheses regarding how reversible spliceosome phosphorylation contributes to both diel plant cell regulation and abiotic stress adaptation in plants.

**Supplementary Information:**

The online version contains supplementary material available at 10.1186/s12870-022-03870-9.

## Introduction

Alternative splicing (AS) is a fundamental process involved in diversifying the cellular repertoire of mRNA transcripts in order to post-transcriptionally regulate gene expression [1]. It can alter the exon composition of an mRNA transcript to produce multiple transcript isoforms per gene; each with the possibility of being translated into unique proteoforms with specific biological functions, activities, or subcellular localizations [2]. Alternatively, it can involve full or partial intron retainment—a phenomenon commonly found in plants [3, 4], which can result in premature termination codons, producing aberrant transcripts that are targeted by non-sense mediated decay (NMD) [5].

In humans, nearly 95 % of all intron containing genes undergo AS [6], while in the plant model organism—*Arabidopsis thaliana (At)*—recent estimates suggest between 60 – 80 % intron-containing genes are alternatively spliced [7–9]. In humans (Hs), mis-regulation of AS has been associated with 15 % of genetic diseases [10], while in plants, several studies have demonstrated the biological importance of AS in plant metabolism [11] and plant development [12–14], including seed germination [15] and flowering [16, 17]. AS in plants has also been reported to occur in response to abiotic stress such as drought [18], heat [19], salt [20], and under abscisic acid (ABA) signaling [21, 22], in addition to having broad ranging circadian plant cell regulation implications [23, 24]. Collectively, studies implicate RNA splicing as a critical survival mechanism under abiotic stress and correspondingly, a means by which to potentially enhance climate resilience and other important agronomic traits in crops.

AS is performed by a large nuclear-localized, multi-subunit protein complex called the spliceosome [25]. The highly orchestrated assembly of the spliceosome involves the ordered interaction of small nuclear RNAs (snRNAs) and ribonucleoproteins (RNPs) which together bind to form small nuclear ribonucleoprotein (snRNP) complexes: U1, U2, U4, U5 and U6 [26]. Spliceosome formation and splice site recognition is then facilitated by several splicing factors including members of the SERINE/ARGININE-RICH (SR) proteins [27]. SR proteins are one of the best-characterized non-snRNP proteins in the spliceosome. They are found to possess diverse roles in transcript splicing, such as the recruitment and promotion of snRNP binding to active splicing events, and aiding in splicing catalysis by recognizing and selecting splice sites for constitutive and alternative splicing [28, 29]. SR proteins are defined by a highly conserved N-terminal RNA binding domain and a C-terminal arginine-serine-rich (RS) domain [30], which is the target of reversible phosphorylation [31].

Illustrated by the large number of phosphoproteomic studies and plant protein post translational modification (PTM) repositories reporting extensive phosphorylation of SR proteins and snRNPs [32], PTM viewer [33] https://www.psb.ugent.be/webtools/ptm-viewer/), qPTMplants [34] http://qptmplants.omicsbio.info/, the plant spliceosome and its regulatory proteins (e.g. SR proteins) are a major targets of regulatory phosphorylation events. The phosphorylation and dephosphorylation of RS domains can alter the ability of SR proteins to interact with other splicing related proteins and with RNA, which in turn modifies the pre-mRNA splicing programme [35]. Based on their orthology to known human splicing-related kinases, these phosphorylation events are likely catalyzed by three protein kinase families: the SERINE/ARGININE PROTEIN KINASES (SRPKs), ARABIDOPSIS FUS3 COMPLEMENT (AFC) or Pre-mRNA PROCESSING FACTOR 4 (PRP4Ks) protein kinases.

In humans, the phosphorylation activity and function of the three splicing-related kinase families have been well-established. HsSRPK1 phosphorylates the N-terminal half of the RS domain of SR proteins [36–38], while CDC-Like kinase 2 (CLKs), the human AFC orthologs, further phosphorylate SR proteins to generate hyper-phosphorylated SR proteins that then initiate early spliceosome assembly [39]. Alternatively, HsPRP4K phosphorylation activity is required to stabilize snRNP association during spliceosome assembly [40]. However, it is currently unknown if these human splicing-related kinase families parallel in function to those in plants. With plants demonstrating unique functional differences in their AS landscapes compared to metazoans [9], it is likely that the expansion of splicing-related kinase families in plants parallels the need for additional regulation of diverse plant specific processes.

Our understanding of how AS impacts multiple facets of the plant cell environment has begun to be explored at the transcriptional-level, however, how these changes affect translational and post-translational outcomes (e.g. proteoforms) in plants is only in its infancy. For example, to what extent does the phosphorylation activity of splicing-related kinases affect the outcomes of RNA splicing? Therefore, to better understand their conservation across photosynthetic eukaryotes and to elucidate their roles in plant development and stress adaptation, we performed a genome-scale molecular phylogenetic analysis of the three splicing-related kinases families. We then combine this with transcriptomic and bioinformatic analyses of *A. thaliana* splicing-related kinases to infer their connection to specific biological processes where RNA splicing has been shown to be significantly altered within photosynthetic eukaryotes, specifically diel plant cell regulation and abiotic stress response. Overall, our findings provide a foundational understanding of these potentially important protein kinase families, opening up numerous avenues for future fundamental and applied plant research.

## Results and Discussion

### Phylogenetic and evolutionary relationships between photosynthetic eukaryote splicing kinases

**Fig. 1:**
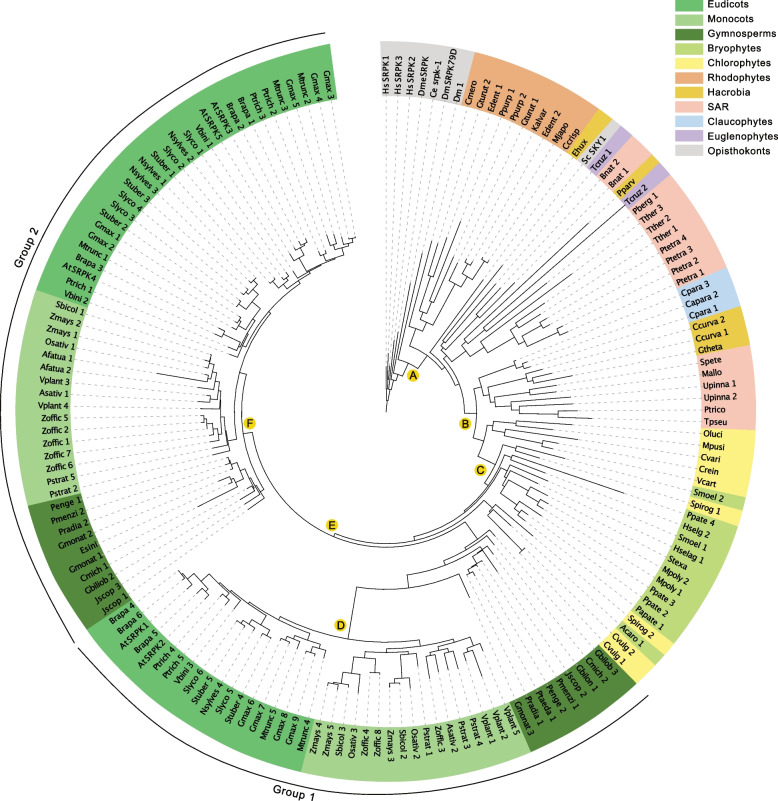
Maximum likelihood phylogenetic tree of SRPK kinases across unicellular and multicellular photosynthetic and select non-photosynthetic organisms. Key nodes are labelled with branch support values from maximum likelihood interference (IQTree), bayesian (Mr. Bayes) and an additional maximum likelihood interference (PhyML), respectively. Node A: (0.97, 1.00, 0.95); Node B (0.98, 0.99, 0.97); Node C: (1.00,1.00, 0.99); Node D: (1.00, 1.00, 1.00); Node E: (1.00, 1.00, 0.80); Node F: (1.00, 0.99, 0.99)

**Fig. 2:**
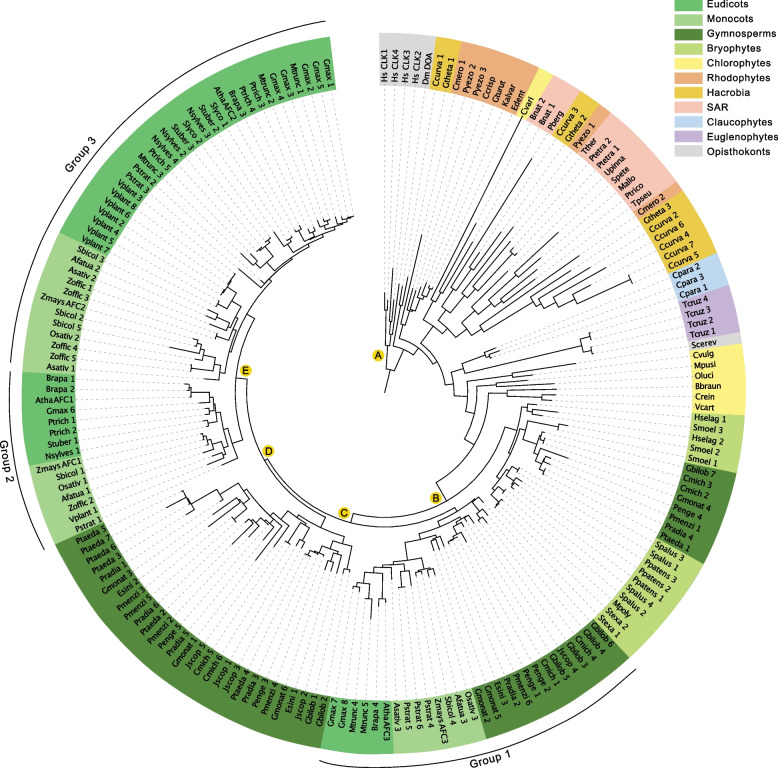
Maximum likelihood phylogenetic tree of AFC kinases across unicellular and multicellular photosynthetic and select non-photosynthetic organisms. Key nodes are labelled with branch support values from maximum likelihood interference (IQTree), bayesian (Mr. Bayes) and an additional maximum likelihood interference (PhyML), respectively. Node A: (1.00, 1.00, 1.00); Node B (1.00, 1.00, 0.99); Node C: (0.97, 1.00, 0.96); Node D: (0.88, 0.93, 0.78); Node E: (0.99, 1.00, 0.99)

**Fig. 3:**
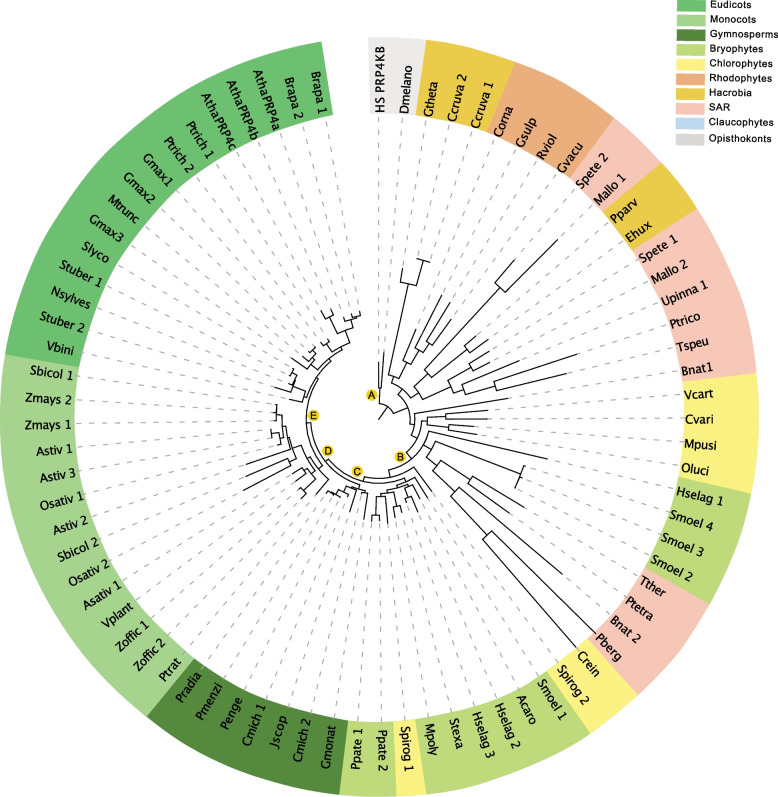
Maximum likelihood phylogenetic tree of PRP4K kinases across unicellular and multicellular photosynthetic and select non-photosynthetic organisms. Key nodes are labelled with branch support values from maximum likelihood interference (IQTree), bayesian (Mr. Bayes) and an additional maximum likelihood interference (PhyML), respectively. Node A: (1.00, 1.00, 1.00); Node B: (0.77, 0.78, 0.80); Node C: (0.86, 1.00, 0.90); Node D: (0.98,0.86, 0.95); Node E (0.98, 0.56, 0.96)

To determine the evolutionary history of the SRPK, AFC, and PRP4K protein families, we constructed phylogenetic trees from a taxonomically diverse set of photosynthetic and non-photosynthetic eukaryotes using a combination of maximum-likelihood and Bayesian analysis approaches (Figs. 1, 2 and 3). Correspondingly, our classification of SRPK, AFC, and PRP4K splicing-related kinases was derived from metazoan orthologs (included here), which are known to impact spliceosome function [40–42]. Overall, we see an expansion of each splicing-related kinase family in photosynthetic eukaryotes over evolutionary time, culminating with the emergence of a family organization in land plants that indicates potential diversification of their biological and cellular functions with the colonization of land by plants.

#### SRPK-family

Interestingly, our molecular phylogenetic analysis revealed a discrepancy between the number of protein members in an organism and that organism’s evolutionary position. For example, early unicellular photosynthetic eukaryote *Cyanophora paradoxa* possesses three SRPK proteins (Fig. 1, Supplemental Table 1), while other organisms appearing later in evolutionary time such as, *Volvox carteri* have only one SRPK member. This was also noted by Giannakouros and colleagues [43], who found no one-to-one SRPK gene corresponding sequence between evolutionary distant species, including the three SRPK genes of *Drosophila melanogaster*, which were found to not equate to any one of the three human SRPK genes [44]. These findings suggest independent SRPK duplication events throughout the evolution of distant species resulting in multiple unique SRPK copies in certain taxa. It is possible that this diversification is partially a result of increasing organismal complexity coupled with increased integration of RNA splicing as a regulatory mechanism. Highlighting this hypothesis is HsSRPK1, which is involved in the signal transduction of epidermal growth factors (EGF) through the protein kinase B (Akt) pathway to phosphorylate SR proteins and initiate the downstream pre-mRNA splicing needed for cellular growth [41]. Similarly, Drosophila SRPKs are necessary for the formation of oocyte microtubule spindle assembly and karyosome formation which is critical for proper meiotic division [45].

Despite the disconnect between evolutionary scale and SRPK copy number, there seems to be two SRPK groups that emerge with the evolution of spermatophytes (Fig. 1). Using Arabidopsis as the benchmark, we see AtSRPK1 and AtSRPK2 as part of ‘Group 1’ SRPKs, while AtSRPK3, AtSRPK4, and AtSRPK5 form ‘Group 2’ SRPKs. This suggests that: 1) Group 1 and 2 SRPKs may have arisen through duplication event(s) and 2) there is an evolutionary pressure to maintain two distinct SRPK groups in spermatophytes. Observation of 2 SPRK groups in spermatophytes is consistent with gene duplication and subsequent gene-specific functionalization in land plants, something that has also been associated with whole genome duplication events [46, 47]. However, most duplicated genes are subsequently lost, reverting back to a single gene status [48], while duplicated genes whose function impact core eukaryotic processes are often retained, with proteins involved in signaling and metabolism showing a higher post-duplication retention rate [49]. Thus, the SRPK protein family, which is conserved across eukaryotes and whose function lies at the core of RNA processing regulation, are contenders for functional diversification and selective pressure towards maintaining multiple copies. Further, additional pressure to maintain two SRPK groups may result from evolved differences in substrate specificities and tissue specific expression patterns. For example, human and mice SRPK families have been found to be tissue specific [50, 51], with human SRPKs maintaining tissue specific expression patterns dependent on developmental stage, suggesting functional specialization that parallels organism multicellularity and complexity [52]. Therefore, spermatophytes likely maintained two SRPK groups for their distinct substrate specificities that benefit multicellular functioning.

Alongside our extensive examination of photosynthetic eukaryotes, our study includes select opisthokonts for outgroup comparison. Here, we find the *Saccharomyces cerevisiae* (budding yeast) SRPK homolog (SRPK1-like Kinase; SKY1) to be most related to those found in photosynthetic eukaryotes, particularly euglenophytes, stramenopiles / heterokonts, alveolates, and rhizaria (SAR) phylogenetic groups (Fig. 1). However, SKY1 contains a glutamate instead of a highly conserved glutamine at position E569 [53]. Interestingly, this residue change may be due to the yeast genome lacking SR protein encoding genes and a near lack of AS in budding yeast [54], implying that SKY1 is a paralog rather than an ortholog of SRPKs. Similarly, the yeast AFC homolog does not group with opisthokonts, advancing the hypothesis that these splicing-related kinases may have different functions in yeast (Fig. 2). Interestingly, our phylogenetic search did not yield a yeast PRP4K ortholog (Fig. 3), however, many of the organisms selected for our phylogenetic search did not have PRP4K ortholog, including rhodophytes with only a few exceptions (Supplemental Figure 1).

#### AFC-family

Here we found that AFC kinases form three distinct phylogenetic groups (Fig. 2). Using *Arabidopsis* AFCs as representative proteins, we find AtAFC1 and AtAFC2 to divide into separate groups at the level of monocots and eudicots, while AtAFC3 diverges earlier at the gymnosperm level, suggesting that Group 3 AFCs may be more basal in spermatophytes. Given the formation of these distinct AFC groups across the diverse photosynthetic eukaryotes sampled here, it is probable that AFCs possess specific functions in plants, with AFC1 and AFC2 likely performing cellular and/or biological functions unique to angiosperms.

#### PRP4K-family

Curiously, unlike the SRPKs and AFCs, we do not find the PRP4Ks to separate into distinct phylogenetic groups, rather clustering at the Brassicaceae family level (i.e. *Arabidopsis* and *Brassica rapa*), indicative of more recent gene duplication events. We find this phenomenon as far back as liverworts and other early land plants, while green algae and other photosynthetic secondary endosymbionts (e.g. *Emiliania huxleyi*) possess only a single PRP4K protein. This suggests that with migration to land, PRP4Ks in photosynthetic eukaryotes have undergone gene duplication events that have seemingly not resulted in orthologs with specialized cellular or biological functions. Alternatively, this may indicate that PRP4Ks perform critical regulatory functions related to RNA splicing in land plants that requires a certain level of genetic redundancy. Further, a third PRP4K (PRP4Kc) seems unique to *Arabidopsis* since *Brassica rapa*, a close relative to *Arabidopsis*, possesses only two PRP4K proteins (Supplemental Table 1), suggesting that PRP4Ks may have been further duplicated in *Arabidopsis*.

### Conservation of protein domain composition across splicing-related kinases

Next, to define gene-function relationships, we endeavored to better understand if protein domain organization parallels our phylogenetic relationships, as it has been found that ortholog identification can be clouded by insertion, deletion, and shuffling of domain architecture [55, 56]. To assess this, we performed a comprehensive domain analysis of the three splicing-related kinase families using a new domain meta-analysis tool called DomainViz [57]. By using DomainViz we were able to deduce the positionality and conservation of protein domains in each splicing-related kinase family.

#### SRPK-family

**Fig. 4:**
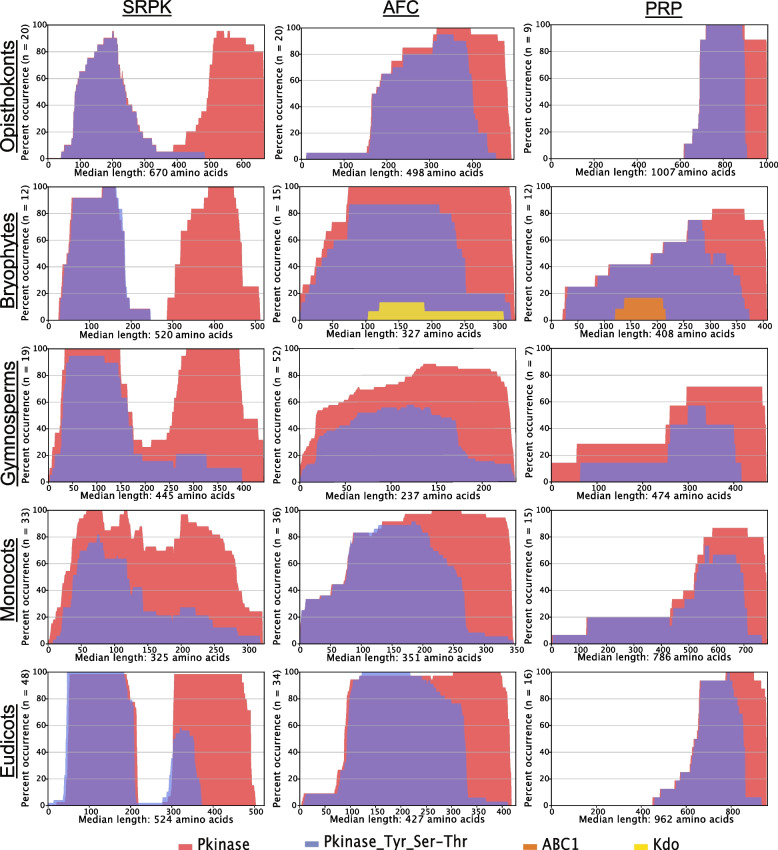
Comparative phylogenetic analysis of conserved domain across phylogenetics groups: opisthokonts, bryophytes, gymnosperms, monocots, and eudicots. Positionality and length of domain is displayed on the x-axis, while the y-axis represents number of organisms whose peptide sequence contains the identified domain. Domain prediction was acquired through PFAM using DomainViz (http://uhriglabdev.cirrus.ualberta.ca/domainviz; [57])

Here we uncovered a prominent spacer region located midway through the peptide sequence (Fig. 4). This spacer region bifurcates the kinase domain into two halves creating a bipartite kinase domain, and is specific to the SRPK family. The bipartite kinase domain is present across both unicellular and multicellular eukaryotic organisms suggesting that the evolution of the spacer domain occurred early in the formation of the SRPK protein kinase family and is likely important for protein function. In mammals, the SRPK spacer region functions in localizing the protein to the cytoplasm, and removal of the spacer domain increases SRPK translocation to the nucleus, resulting in the hyper-phosphorylation of SR proteins and initiation of splicing reactions [58]. More precisely, the spacer domain functions as a docking motif by anchoring the SRPK protein to the Hsp70/Hsp90 machinery and thereby restricting SRPK to the cytoplasm [59]. More recent findings suggest that the spacer domain promotes appropriate activation loop folding thus bridging the two catalytic kinase domains into proximity and facilitating the formation of an active conformation [60–62]. Interestingly, we found that monocot SRPKs largely lack this spacer region (Fig. 4). Based on linker deletion studies in mammals, this suggests that a sub-population of monocot SRPKs possess a more predominant nuclear localization relative to other photosynthetic eukaryotes and therefore a potentially stronger impact on RNA splicing outcomes.

#### AFC-family

Unlike SRPKs, the AFC kinase domain is present as a singular unit (Fig. 4). In the phylogenetic groups of monocots, eudicots, and opisthokonts we observed the introduction of a polypeptide extension at the N-terminus with an unspecified function which gives the appearance of a kinase domain shift towards the C-terminus. Interestingly, a small subset of hacrobia AFCs (approximately 10 %) uniquely possesses a RIO1 domain (PF01163) situated in the middle of the protein sequence (Supplemental Figure 1). RIO1 domains are derived from a family of serine kinase domains found in archaea, bacteria, and eukaryotes [63]. In *S. cerevisiae*, RIO1 is vital for proper cell cycle progression and processing ribosomal RNA [64], which indicates an uniquely adapted function for these AFCs in hacrobia organisms. Similarly, approximately 15 % of chlorophyte protein sequences contain a sulfotransferase-1 domain (PF00685) along with a small percentage of bryophytes possessing a Kdo domain (PF06293) (Fig. 4, Supplemental Figure 1). Both sulfotransferase-1 and Kdo domains have structural similarity to kinase domains [65], which likely highlights some unique structural elements to the AFC kinase domains of chlorophytes and bryophyte AFCs, respectively.

#### PRP4K-family

Paralleling AFC kinases, the kinase domain of PRP4Ks appears to shift towards the C-terminus in land plants due to the presence of an undefined peptide region in the N-terminal region of the peptide sequence (Fig. 4). We also observe a substantial increase in overall PRP4K protein length along the same evolutionary axis, with eudicots and opisthokonts possessing notably longer PRP4K proteins relative to earlier photosynthetic eukaryotes. Interestingly, almost 20 % of PRP4K proteins in bryophytes contain an atypical protein kinase domain called ABC1 located towards their N-terminus. Similar to the AFCs, this likely indicates additional structural complexity in the kinase domains of PRP4Ks. Proteins maintaining similar ABC1 domains in yeast [66] and *Escherichia coli* [67] were found to be dually localized to the nucleus and mitochondria, suggesting that some bryophyte PRP4Ks may have unique and unconventional biological functions. Overall, the high degree of domain conservation among phylogenies of the splicing-related kinase families in photosynthetic eukaryotes supports our molecular phylogenetic conclusions and that splicing-related kinase domain conservation is maintained across the domains of life.

### Cis-regulatory element motifs of the splicing-related kinases

With protein domain architecture largely conserved within each splicing-related kinase family, we next aimed to define biological and cellular function relationships through a bioinformatic analysis of the *cis*-regulatory element (CREs) composition of each splicing-related kinase family. Given the lack of known functions for splicing-related kinases in plants, examination of CREs was logical for elucidating functional differences within, and between, families of splicing-related kinases, since CREs play a major role in regulating gene expression. Due to the lack of CRE information across species, we utilized the *Arabidopsis* SRPK, AFC, and PRP4K kinases as representative genes for this analysis. To extract CREs for the *Arabidopsis* splicing-related kinase genes, we used AtCisDB [68], which contains predicted and experimentally derived CREs present in the deduced promoter regions of *Arabidopsis* gene sequences. With *Arabidopsis * representing the best characterized plant system to date, it represents a reliable proxy for relating CRE information to gene / protein function(s).

**Fig. 5:**
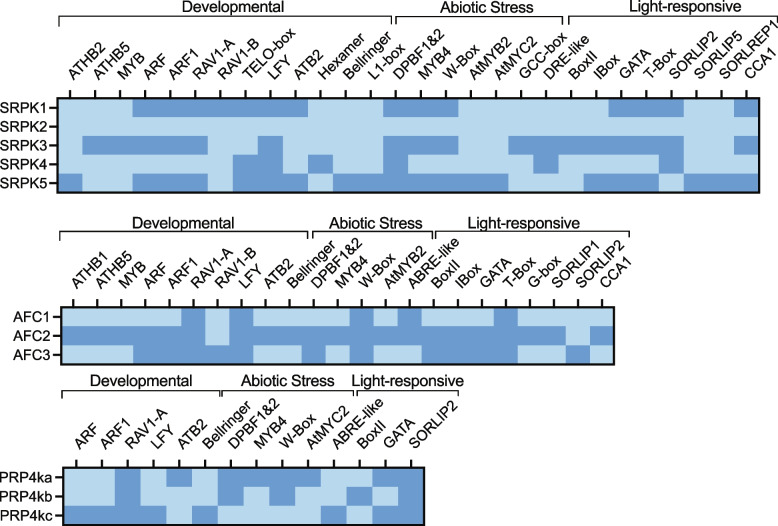
Identification of putative *cis *regulatory elements (CREs) on the gene sequence of SRPKs, AFCs, and PRP4Ks. Presence of CREs is denoted by dark blue while absence is denoted by light blue. Data was acquired by mining AtCisDB database (https://agris-knowledgebase.org/AtcisDB/; [68])

Here, we find a collection of established CREs that are *SRPK* group-specific and group non-specific (Fig. 5). This includes meristematic growth related CREs, such as TELO-box, BELLRINGER (BLR), LFY, and L1-box across the *SRPK*s, suggesting a critical role in plant development (Fig. 5). We suspect that there may be substrate specificity differences between Group 1 and 2 SRPKs, that when coupled with CRE commonalities, offers a means by which to deploy the SRPK complement needed to initiate particular splicing patterns.

Despite being divergent phylogenetically, members of the AFC family share a common subset of CREs that include: T-box, W-box, LFY, and RAV1-A (Fig. 5). The presence of a W-box in all *AFC* promoter sequences indicates the core involvement of all AFC family members in pathogen attack and abiotic stress response. The W-box promoter sequence is responsible for the expression of pathogen defense related genes across multiple plant species [69–71], while more recent descriptions of the W-box CRE demonstrated a role in the down-regulation of genes induced by heat and salinity stress [72]. Of the *AFCs*, *AFC2* possesses a comparatively large number of CREs, suggesting that AFC2 may performing the majority of AFC-mediated spliceosome regulation in land plants. Overall splicing-related protein kinases share promoter sequences predominantly involved in developmental and abiotic stress pathways.

### Transcriptional expression patterns of the splicing-related kinases indicate diversification of biological and cellular responses

In plants, RNA splicing has been investigated for its role in a number of biological and cellular processes. This has included: abiotic stress responses [9, 73, 74], development [75, 76], and diel plant cell regulation [77]. These disparate studies have involved examining cold [78], heat [79, 80], osmotic [20] stress responses, along with developmental traits such as flowering [81] and the circadian clock [82]. Each of these studies has revealed RNA splicing to be a central element in plant cell regulation and overall plant biology. Correspondingly, we next mined well-established, publicly available gene expression datasets, such as Genevestigator ([83], https://genevestigator.com/) and ePlant ([84], https://bar.utoronto.ca/eplant/), in addition to performing NanoString transcriptomic analysis of all *Arabidopsis* splicing-related kinases to elucidate their gene expression dynamics in response to plant development, abiotic stress response, and diel plant cell regulation.

### Developmental expression

From our CRE analysis, we found specific splicing-related kinases within each family to possess promoter sequences related to plant development, such as BLR, LFY, and RAV1-A promoter sequences. These CREs are associated with genes involved in a multitude of developmental processes such as flowering (Fig. 5), with flowering impacted by the AS of *CIRCADIAN CLOCK ASSOCIATED 1* (*CCA1*), a core circadian clock gene [85]. Mammalian SRPK family members exhibit tissue-specific expression profiles characteristic of specialized functions required for multicellular development [50, 86]. Correspondingly, it is possible that the functional diversification of the plant splicing-related kinases is driven by their tissue / organ expression profiles that differ according to developmental time. Therefore, in order to dissect the roles of these splicing-related kinases in plant development, we analyzed transcript expression levels of the Arabidopsis splicing-related kinases at major developmental stages using gene expression data from ePlant and Genevestigator.

#### Cell differentiation & organ development

**Fig. 6:**
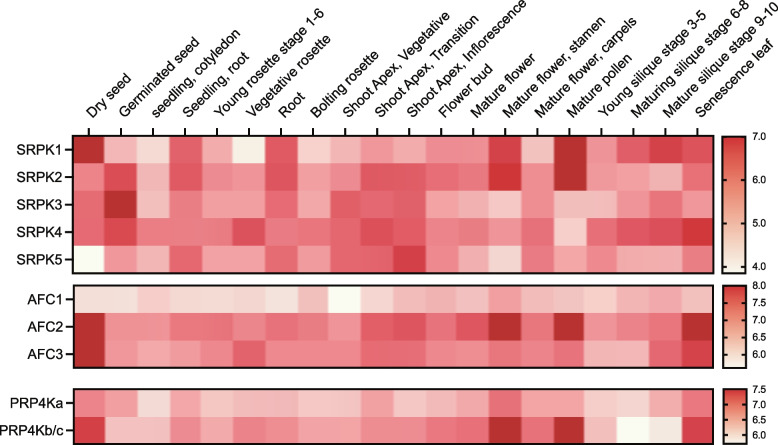
Relative transcript abundance at various stages of Arabidopsis development, from seed to senescence. Values were acquired from BAR ePlant and absolute values were log_2_ transformed (https://bar.utoronto.ca/efp/cgi-bin/efpWeb.cgi; [94])

We find numerous developmental related CREs across the *Arabidopsis SRPK*, *AFC*, and *PRP4K* families (Fig. 5). For example, the TELO-box CRE, present in the promoter regions of *AtSRPK1*, *AtSRPK4*, and *AtSRPK5*, is associated with translation-related genes in root meristems such as *eukaryotic elongation factor 1 alpha (eEF1A)* and several ribosomal protein genes [87–89]. *eEF1A* is expressed in the dividing cells of the root primordia to assist with cytoskeleton formation in germinating seeds, embryos, shoot and root meristems [90]. *AtSRPK1* (Log_2_ seedling root expression = 6.39, Log_2_ mature root expression = 6.47), *AtSRPK2* (Log_2_ seedling root expression = 6.48, Log_2_ mature root expression = 6.53), and *AtSRPK5* (Log_2_ seedling root expression = 6.33, Log_2_ mature root expression = 6.28) are all highly expressed in the roots (Fig. 6), implicating these AtSRPKs in cell expansion. Alternatively, *AtSRPK3* is highly expressed during seedling germination (Log_2_ = 7.32), indicating expression in the early stages of organ growth and development. Similarly, the L1-box, present only in the *AtSRPK5* promoter, is associated with genes controlling the growth and development of the outermost layer (L1) of the shoot apical meristem [91]. We also find *AtSRPK2*, *AtSRPK3*, *AtSRPK4*, and *AtSRPK5* to increase in expression at the shoot apex from the vegetative to inflorescence stage, suggesting that these SRPKs may be involved in cell differentiation (Fig. 6; Supplemental Table 2). The potential involvement of AtSRPKs in cell differentiation and organ development coincides with crucial roles for HsSRPKs in neurodevelopment [92] and in catalyzing the life-beginning event of parental genome reprogramming in the fertilized oocyte [93].

All members of the *AtAFC* and *AtPRP4K* families have a RAV1-A promoter which is involved in the development of rosette leaves and lateral roots (Fig. 5) [95]. *AtPRP4Ks* demonstrate increased expression in the young rosette (Log_2_ _*AtPRP4Ka* = 6.38, Log_2_ _*AtPRP4Kb/c* = 6.67), vegetative rosette (Log_2_ _*PRP4Ka* = 6.48, Log_2_ _*AtPRP4Kb/c* = 6.95), seedling (Log_2_ _*AtPRP4Ka* = 6.69, Log_2_ _*AtPRP4Kb/c* = 6.90) and mature root (Log_2_ _*AtPRP4Ka* = 6.52, Log_2_ _*AtPRP4Kb/c* = 6.89) developmental stages (Fig. 6). *AtAFC2* also shows expression increase during young rosette (Log_2_ = 7.41), vegetative rosette (Log_2_ = 7.23), seedling (Log_2_ = 7.36) and mature root (Log_2_ = 7.40) developmental stages (Fig. 6). Interestingly, HsCLKs, the AFC human orthologs, phosphorylate SR splicing factors necessary for the Wnt pathway in human mesenchymal stem cells, which plays a central role in organogenesis, cell differentiation, and tissue remodeling [96, 97]. CLKs have also been implicated in the AS regulation of *HMGA2*, a gene required for human hematopoietic stem cell development [98]. Correspondingly, this positions AFC splicing-related kinases as critically important developmental regulators.

#### Flowering & seed maturation

We find several flowering related CREs in the promoters of *AtSRPK* genes (Figs. 5 and 6). BLR (present in the *AtSRPK5* promoter) and LFY (present in the *AtSRPK1, AtSRPK4,* and *AtSRPK5* promoters) in particular, are associated with floral genes that drive floral organ development [99, 100]. Correspondingly, these CREs align with high *AtSRPK* gene expression in reproductive developmental stages beginning at the shoot apex inflorescence through to dry seed (Fig. 6, Supplemental Figure 2, Supplemental Tables 2 and 3). On a whole, *AtSRPK*s have reduced expression in mature flower, with only a marginal increase at bolting (Supplemental Figure 2, Supplemental Table 3). However, when we contrast this with their expression levels within individual floral organs, we find exceptionally high, organ-specific *AtSRPK1* and *AtSRPK2* expression in the stamen (Log_2_ _*AtSRPK1* = 6.77, Log_2__*AtSRPK2* = 6.96) and pollen (Log_2_ _*AtSRPK1* = 9.34, Log_2_ _*AtSRPK2* = 9.60), suggesting that SRPKs may be required for the initiation of reproductive related AS events.

PRP4Ks may also be involved in developmental processes due to the presence of auxin response factor CREs (ARF and ARF1) present in the *AtPRP4Kc* promoter. ARFs have been implicated in development through loss-of-function mutant analysis [101–103], with ARF1 controlling leaf senescence and floral organ abscission in *Arabidopsis* [104]. Interestingly, *AtAFC2*, *AtAFC3*, and *AtPRP4K*s also have high expression in the male associated flower organs (Fig. 6, Supplemental Table 2). Further, Kanno and colleagues (2018) found that *prp4ka* mutant plants possess delayed flowering, implicating PRP4Ka in flower organ development. The same group reported that the *prp4ka prp4kb* double mutant was not viable, emphasizing their integral involvement in plant reproduction.

Beyond organ flower development, it seems select splicing-related kinases (*AtSRPK1*, *AtSRPK4*, *AtAFC2*, *AtAFC3*, and *AtPRP4Ka*) may be involved in silique maturation. Expression levels increase steadily throughout silique maturation culminating with peak expression levels in dry seeds (Fig. 6). *AtSRPK1* is particularly elevated at all stages of silique maturation and has the highest expression level in dry seeds (Supplemental Table 2). Similarly, *AtAFC2*, *AtAFC3*, and *AtPRP4Kb/c* each have considerable increases in dry seed expression (Log_2_ = 8.59, Log_2_ = 8.17, Log_2_ = 7.39, Log_2_ = 7.39, respectively) relative to other tissues (Fig. 6). Interestingly, recent transcriptome profiling of dry seeds found that while overall transcription declined in dry seeds, AS increased [105]. This is specifically highlighted by AS regulation of *PHYTOCHROME INTERACTING FACTOR 6* (*PIF6*), whose AS variant demonstrates reduced seed dormancy [106].

#### Senescence

Following silique maturation, we find select splicing-related kinases (*AtSRPK1*, *AtSRPK4*, *AtAFC2*, *AtAFC3*, and *AtPRP4Kb/c*) to possess increased expression at senescence (Fig. 6, Supplemental Figure 2, Supplemental Tables 2 and 3). In particular, *AtSRPK4*, *AtAFC2* and *AtPRP4Kb/c* sharply rise in their expression at senescence. In poplar trees (*Populus tomentosa*) a splice variant of the NAC transcription factor PtPD26 was found to regulate numerous other NAC transcription factors which delay leaf senescence [107]. We suspect that these splicing-related kinases play a role in cell cycle progression or cell death through the phosphorylation of splicing factors required for the splicing of senescence related genes. Currently, our understanding of the extent to which AS plays a role in development remains largely unresolved, however, our data indicates that future experimentation should focus on elucidating the role these specific splicing-related kinases play in modulating plant death.

### Abiotic stress

Abiotic stresses such as drought, salt, heat, and cold, demand accurate and rapid transcriptional modulation for successful adaptation by plants. Transcriptomic studies examining these stresses have found extensive global transcriptome changes in response to osmotic, salt, heat, and cold stresses that occur within minutes to hours of induced stress. For example, 42 %, 46 %, and 53 % of the Arabidopsis transcriptome had a greater than two-fold change from 3 to 27 h of 4 ºC, 200 mM mannitol, and 100 mM NaCl stress, respectively [108]. Previous work by Calixto and colleagues (2018) reported rapid global transcriptional change and AS in response to cold stress of which many were splicing factors and other RNA binding proteins. Such rapid transcriptional change is likely to include fluctuating activities of upstream regulators such as splicing-related kinase*.* Hence, we sought to investigate the potential involvement of splicing-related kinases in abiotic stress response using ePlant and Genevestigator databases, in addition to acquiring new transcriptome data as part of this study. To do this, we quantified the expression of all 11 splicing-related kinases in either the shoots or roots under osmotic, salt, heat, and cold stress using experimental conditions that parallel those tested in Kilian et al., 2007. Correspondingly, we subjected *Arabidopsis* seedlings to 300 mM mannitol to simulate osmotic stress and 150 mM NaCl for salt stress, while heat stress involved a 38 ºC exposure for 3 h followed by a 3 h recovery, which is the time at which the majority of splicing-related kinase genes experienced the highest transcriptional changes (Supplemental Figures 3, 4 and 5, and Supplemental Table 4). For cold-stress treatments, *Arabidopsis* seedlings were exposed to 4 ºC for 24 h.

#### Osmotic & salt stress

**Fig. 7:**
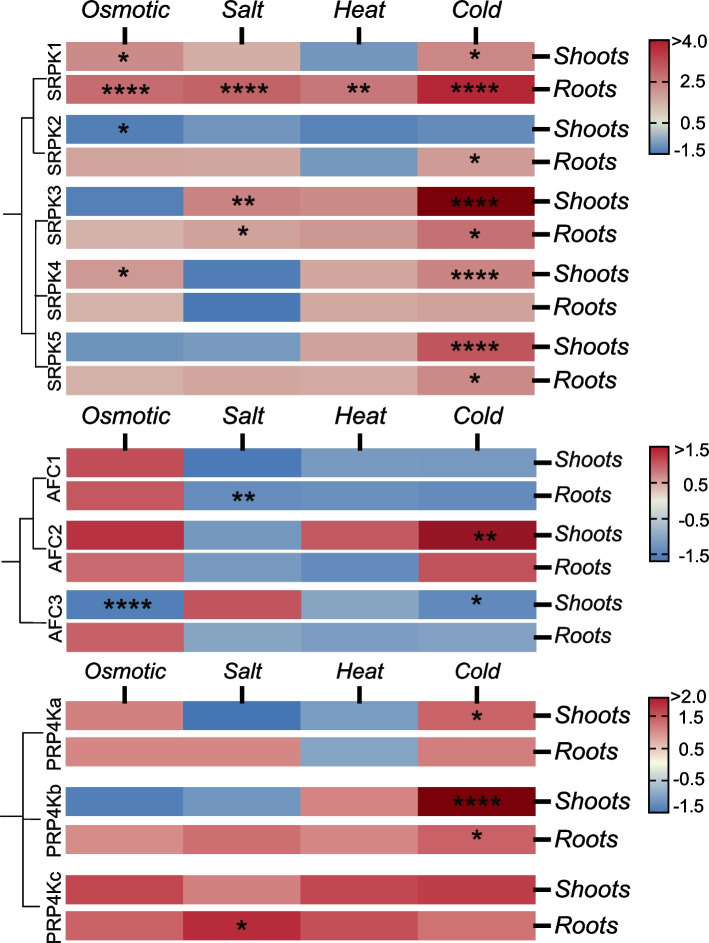
Relative Log_2_ fold change of splicing-related kinases transcript abundance under abiotic stresses: osmotic, salt, heat, and cold stress. Each stress was induced with parallel parameters from Kilian et al., 2007. 4 replicates were averaged and the comparison to control were FDR adjusted *p*-values

Both *AtSRPK1* and *AtSRPK5* possess CREs indicating involvement in drought-related response pathways due to their shared DPBF1&2, MYB4, and ATB2 promoter sequences, all of which have been shown to regulate the expression of genes related to drought and in the abscisic acid (ABA) mediated response pathways [109, 110]. Correspondingly, *AtSRPK1* exhibited a significant increase in expression under both salt and osmotic stress (Fig. 7). *AtSRPK3* also maintained a significant increase in root (Log_2_FC = 1.26, *q-value* ≤ 0.02) and shoot (Log_2_FC = 1.88, *q-value* ≤ 0.01) expression under salt stress (Fig. 7, Supplemental Figure 3, Supplemental Tables 4 and 5). We also see a significant increase in *AtSRPK4* expression in shoots (Log_2_FC = 1.44, *q-value* ≤ 0.03) upon osmotic stress, while *AtSRPK2* is the only *AtSRPK* that significantly decreases in shoot expression under osmotic stress (Log_2_FC = -1.37, *q-value* ≤ 0.02). Interestingly, despite *AtSRPK5* possessing a number of drought-related promoter sequences (Fig. 5), we found no significant change in its expression relating to osmotic and salt stress, suggesting that *AtSRPK5* induction in response to drought may occur at specific stages of plant development or in specific organs not sampled here.

Both the *AtAFC* and *AtPRP4K* families also possess a handful of ABA-related CREs (Fig. 5). For example, the promoters of *AtAFC1* and *AtAFC2* have an ABA-responsive element (ABRE-like) CRE, which is involved in osmotic stress response [111], while *AtAFC2* and *AtAFC*3 both maintain a Dc3 promoter-binding factor (DPBF1&2) CRE, which is ABA and water stress response related [109, 112]. Despite the presence of these drought-stress related CREs, we find that *AtAFC*s generally decrease in expression under salt conditions and increase their expression under osmotic conditions (Fig. 7). In particular, *AtAFC1* significantly decreases (Log_2_FC = -1.38, *q-value* ≤ 0.01) under salt stress in the roots, while *AtAFC3* significantly decreases (Log_2_FC = -1.57, *q-value* ≤ 0.001) under osmotic stress in the shoots. *AtPRP4K*s also have drought-stress related CREs, such as RAV1-A, DPBF1&2, and W-box (Fig. 5), but were not significantly influenced by any of the stressors applied in our study outside of *AtPRP4K*c under salt conditions (Root_Log_2_FC = 1.83, *q-value* ≤ 0.05) (Fig. 7, Supplemental Table 6). This finding contradicts the previous hypothesis that *AtPRP4Kc* is a pseudogene [14], indicating that *AtPRP4Kc* is an expressed gene that has specific roles in abiotic stress response. It is possible however, that the transcriptional changes in other *AtPRP4K* genes were not captured by our harvesting strategy as it was end-point based.

#### Heat stress

Although we did not specifically find heat-related CREs on any of the splicing-related kinases (Fig. 5), we do see a significant impact of heat on *AtSPRK1* expression (Fig. 7). *AtSRPK1* uniquely demonstrates a significant increase (Log_2_FC = 2.1, *q-value* ≤ 0.001) under heat relative to the other *AtSRPK*s. This was unexpected, since the ePlant microarray data found *AtSRPK3* and *AtSRPK4* exhibiting differential expression under heat stress, while *AtSRPK1* remained relatively unchanged (Supplemental Figure 3, Supplemental Table 4). Again, our biological sample size provides a more robust depiction of stress induced changes in expression relative to previous studies. We observe *AtSRPK1* significantly increasing under the majority of the stresses compared to the other members of the AtSRPK family, suggesting that *AtSRPK1* may be specifically required for AS of certain genes central to abiotic stress responses.

HsSRPKs have been classified as “stress kinases” due to their unique position in transmitting cellular stress signals from the cytoplasm to the nucleus through their phosphorylation of SR splicing factors activating their translocation into the nucleus to induce splicing in response to stress signals [60]. For example, sorbitol-induced osmotic stress in mammalian cells resulted in a sufficient osmotic shock to dissociate HsSRPK1 from its chaperone complexes resulting in its translocation from the cytoplasm to the nucleus [113]. Further, upon treatment with paraquat (a compound that induces superoxide formation and oxidative stress) human neuroblastoma cells increase HsSRPK2 nuclear translocation to adjust the splicing pattern of genes involved in DNA repair, cell cycle control, and apoptosis [114]. When combined with the drought expression changes in* AtAFC1* and *AtAFC3* along with *AtPRP4K*, our results suggest that splicing-related kinases, in particular SRPKs, have a broad role in mediating plant drought-like responses.

#### Cold stress

*AtSRPK3* and *AtSRPK4*, both belonging to Group 2 SRPKs, share a dehydration-responsive element (DRE-like) CRE, which is important for the transcriptional regulation of cold-responsive genes [110] and support our hypothesis that there is functional specificity to Group 1 and 2 SRPKs. Although only *AtSRPK3* and *AtSRPK4* have a cold-related CRE, we find that cold stress induced a significant increase in all *AtSRPK*s transcript levels across both shoots and roots except for *AtSRPK2* in shoots (Fig. 7, Supplemental Figure 3). Lack of *AtSRPK2* transcriptional change in response to cold correlates with the overall lack of CREs present in the *AtSRPK2* promoter (Fig. 5). It is likely that *AtSRPK2* is either constitutively expressed or has CREs not captured by the AtCisDB database. As such, *AtSRPK2* is likely a constitutively active Group 1 SRPK member while *AtSRPK1* may be a Group 1 stress-responsive SRPK.

Alternatively, *AtAFC2* and *AtAFC1/3* show opposite shoot expression patterns when exposed to cold treatment. *AtAFC2* significantly increases (Log_2_FC = 1.55, *q-value* ≤ 0.01), while *AtAFC3* significantly decreases (Log_2_FC = -1.42, *q-value* ≤ 0.02). Interestingly, The human HsCLK orthologs of plant AFCs, have been shown to be important thermo-sensors that are required for temperature-responsive AS to adjust the circadian biology of mammals [115]. In mammals, lower body temperatures activates CLKs, resulting in increased phosphorylation of SR proteins. Moreover, AFC orthologs in other animal systems are also temperature sensitive, such as in turtle (*Trachemys scripta*) and fruit fly (*D. melanogaster*), which show reduced CLK protein activity above their preferred living temperatures that corresponds to an AS change mediated by temperature dependent CLK activity [115]. Since AFCs are highly conserved across photosynthetic eukaryotes, coupled with the dynamic change in expression observed here in response to cold, it is likely that AFCs also have a role in temperature perception and acclimation *in planta* as well.

*AtPRP4K* expression patterns were primarily impacted by cold stress (Fig. 7), with both *AtPRP4Ka* (Log_2_FC = 1.32, *q-value* ≤ 0.02) and *AtPRP4Kb* (Log_2_FC = 2.61, *q-value* ≤ 0.01) significantly increasing in their expression in shoots as a result of cold stress. Interestingly, fission yeast PRP4K was discovered as a temperature-sensitive mutant defective in RNA splicing [116, 117], suggesting broader evolutionary roles for PRP4Ks in cold-stress responses. Overall given the lack of divergence amongst orthologous PRP4Ks across land plants, coupled with the lack of CRE differences amongst the *AtPRP4K*s, it is likely that PRP4Ks serve largely redundant roles in relation to abiotic stress response.

### Light regulated expression

Recently, co-transcriptional regulation, such as AS, has been highlighted as a mechanism by which plants regulate their internal circadian clock [118]. Correspondingly, regulators of splicing, such as SRPKs, AFCs, and PRP4Ks, may then be involved in the timing of gene expression for circadian clock function. Firstly, we found that various members of the *Arabidopsis SPRK*, *AFC*, and *PRP4K* families have light-dependent CREs in their promoter regions (Fig. 5). In particular, *AtSRPK1*, *AtSRPK3*, and *AtSRPK4* which possess light responsive CREs such as SORLIP2, GATA, and T-box. Alternatively, *PRP4Ka* has an ATB2 CRE which has been shown to be involved in energy supply and demand, while being regulated by light and hypo-osmolality [119, 120]. As well, all *AtPRP4K* members also maintain RAV1-A and SORLIP2 CRE sequences. SORLIP2 is responsive to signals transmitted by the phytochrome A photoreceptor pathway [121], which in *C. reinhardtii* shows a strong light dose-dependent activation [122]. This suggests that *AtSRPK1*, *AtSRPK3*, *AtSRPK4*, and *AtPRP4K*s may be light-activated as well. Therefore, to study how the presence of light-dependent CREs translates to gene expression changes, we mined DiurnalDB [123] to study diel and photoperiodic expression levels of all 11 splicing-related kinase genes (Supplemental Figure 6, Supplemental Table 5). We then experimentally quantified how the transcript level of these splicing-related kinases change throughout the day under 8:16, 12:12, 16:8 and 24:0 photoperiod conditions by measuring Zeitgeber time (ZT) 6, 11, 18, and 23.

#### SRPK-family

**Fig. 8:**
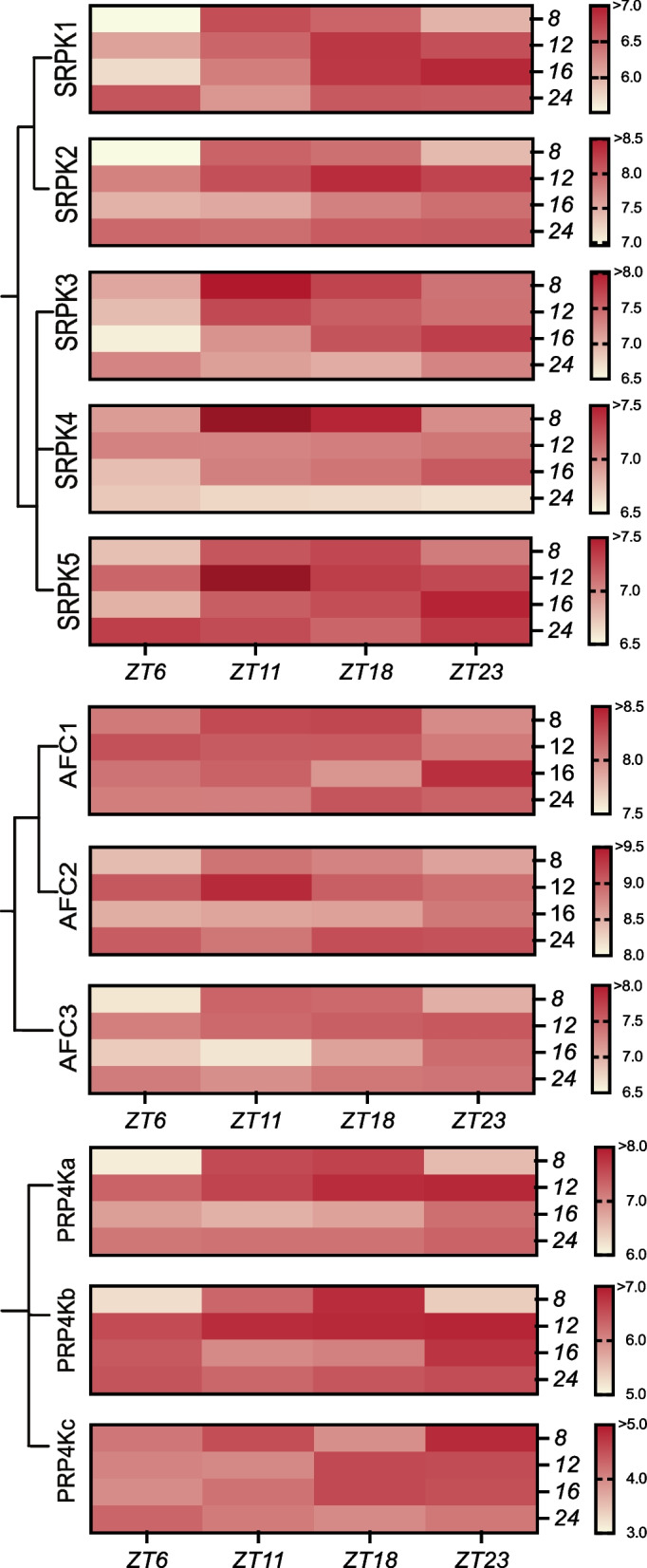
Relative transcript abundance under various photoperiods: 8 h: 16 h, 12 h: 12 h, 16 h: 8 h, 24 h: 0 h. Normalized values were log_2_ transformed and averaged across replicates

Under an 8:16 photoperiod we find that all *AtSRPK*s have a peak expression at mid-day through to the evening (ZT11, ZT18), while under a 12:12 photoperiod, *AtSRPK1* and *AtSRPK2* have a peak expression at ZT18 and ZT23 (Fig. 8; Supplemental Table 7). Under long-day photoperiod conditions (16:8), all *SRPK*s exhibited peak expression at ZT23. Taken together, our SRPK transcript expression data indicates that they may be required for the day-to-night transitions; the same time-point that *CCA1* undergoes AS [85]. Furthermore, the increasing photoperiod shift of *SRPK* peak expression towards the end-of-night (ZT23) suggests that SRPKs may be involved in processes controlled by light signals such as flowering*.*

#### AFC-Family

Similarly, we find that *AtAFC*s maintain peak expression at ZT11 and ZT18 under 8:16 photoperiod (Fig. 8; Supplemental Table 6). The *AtAFC* family also experiences a peak expression shift towards ZT23 upon lengthening of daylight. Since we found various flowering and developmental CREs in the promoters of *AtAFC* genes, they may be involved in diel determined flowering control (Fig. 5). Alternatively, the circadian clock has been shown to be tightly intertwined with age-dependent senescence. *ORE1*, a positive regulator of age induced senescence increases in levels under long day periods and CCA1 directly suppresses *ORE1* delaying senescence [124]. Given that high *AtAFC*s transcript levels are found during leaf senescence (Fig. 6, Supplemental Figure 2), it is possible that *AFC*s are driven by the circadian clock and required for age-dependent senescence.

#### PRP4K-family

Lastly, we see that *AtPRP4K*s possess a peak expression at mid-day (ZT11, ZT18) under 8:16 photoperiod, with *AtPRP4Kc* exhibiting a weak diel expression pattern under all photoperiod conditions (Fig. 8). Like the other splicing-related kinase families, *AtPRP4K*s experience a shift in expression towards ZT23 as day length increases. Collectively, these findings indicate that splicing-related kinases, as a whole, may be highly regulated by light signals.

The significantly increased expression levels of the majority of the splicing-related kinases under cold treatment combined with their elevated expression at end-of-night time-points suggests that they may be involved in regulating temperature dependent AS (Fig. 8). Patterns of clock gene alternative splicing are influenced by changes in photoperiod and abiotic stresses [85], with evidence that AS is involved in the expression of clock gene splice variants at low temperatures [82]. For example, LHY protein abundance is reduced at low temperatures due to the retention of the first intron in the *LHY* 5’-UTR, resulting in NMD [125]. While *CCA1* AS is suppressed by low temperature resulting in an unidirectional production of the *CCA1a* (an intron retention splice variant) that induces freezing tolerance [126]. Furthermore, Filichkin and collogues (2015) showed that SR45 splicing factor may be involved in regulating intron retention of *CCA1*. Initiation of SR45 splicing activity is induced by protein phosphorylation and temperature fluctuations [127]. SR45 phosphorylation may be performed by AFC2, which has been shown to phosphorylate SR45 *in vitro* [128]. Together, this places AFC-family splicing-related kinases as potential regulators clock gene AS. Given the regulatory dominance of the clock over global transcription and the regulatory role of AS in gene expression, it is conceivable that AS may act to bridge between temperature perception with downstream processes [129]. The extent to which regulatory splicing-related kinases, such as the AtSRPKs, AtAFCs, and AtPRP4Ks, are involved in attenuating AS in response to thermo-sensing and photoperiod signals and how these signals change the clock remains to be explored.

### Potential roles for SRPKs beyond RNA splicing

Many of the roles identified for HsSRPKs have been related to RNA splicing of genes involved in developmental and stress response [60, 93, 113, 114]. However, evidence suggests that HsSRPKs have functions beyond RNA splicing [92]. For example, HsSRPK1 has been found to phosphorylate human protamine 1, an arginine-rich protein involved in histone replacement during the development of mature spermatozoa [130]. Additional reports indicate HsSRPKs phosphorylate other non-splicing related, RS-motif containing proteins [43, 131–133]. This raises the possibility that AtSRPKs may have phosphorylation targets other than SR splicing factors and that AtSRPKs may be involved in pathways beyond RNA splicing of abiotic stress or developmental-related genes.

As such, we performed a RS-motif search using the *Arabidopsis* proteome to identify potential non-splicing related proteins that contain canonical RS motif(s) using ScanProsite [92]. A total of 37 RS-motif hits corresponding to a total of 20 proteins were found, with 17 of the 20 identified proteins representing RNA splicing related proteins, and a total of 3 representing non-splicing related proteins (Supplemental Table 9). Of these 3, DEAD-box ATP-dependent RNA helicase 40 (RH40, AT3G06480) is involved in nonsense-mediated mRNA decay and ribosome biogenesis. While Peptidyl-prolyl cis–trans isomerase CYP95 (PPIase CYP95, At4G32420) accelerates folding of proteins by catalyzing the cis–trans isomerization of proline peptide bonds. Both, RH40 and CYP95, are involved in the post-transcriptional processing of mRNA, suggesting that AtSRPKs may be involved in mRNA degradation pathways or mRNA translation.

No consensus RS motif has been established in *Arabidopsis*, however the aforementioned motif was used since it was used to identify RS motif containing proteins in *Mus musculus* proteome [92]. Canonical SR splicing factors proteins were identified using the aforementioned motif search suggesting that this motif is sufficient for identifying RS motif containing plant proteins. However, plants possess divergent SR protein sequences [30], as such, the classification of plant SR proteins does not lie in its specific RS tandem repeat sequences but rather a minimum (20 %) of RS or SR dipeptide composition in the RS domain spanning at least 50 amino acids. Therefore, AtSRPKs may have evolved the capacity to phosphorylate varying RS domains. Future studies will be required to identify the breath of RS sequences phosphorylated by AtSRPKs.

## Conclusion

Post-transcriptional splicing of pre-mRNA can produce unique transcript isoforms that may be required for stress adaptation or for the timing of specific developmental stages, and thus represent an important means by which the cell can fine-tune gene expression [1]. RNA splicing is performed by the spliceosome, whose activity is directed and mediated by SR splicing factors [25]. Upstream regulators of splicing, such as splicing-related kinases, are capable of transmitting external signals to the spliceosome by phosphorylating SR proteins thereby activating their translocation to the nucleus and the initiation of splicing [39, 41]. To date, very few studies have looked at splicing-related kinases’ ability to modulate splicing factor activity and pre-mRNA splicing in plants. Understanding how splicing-related kinases transmit external cues to the spliceosome could provide key insights into the regulatory splicing programme of the plant cell. In this study, we present the first genome-scale analysis of the major splicing-related kinase families of photosynthetic eukaryotes. We find that these splicing-related kinases may have both developmental and abiotic stress related promoter sequences as well as significant expression pattern changes in response to cold, osmotic, and salt stress. Furthermore, the kinase families experience diel expression patterns and shifts upon photoperiod lengthening, suggesting that these kinases may be tightly controlled by light and circadian cues, offering new connections to the timing of critical developmental stages, such as flowering. How splicing kinases in plants impact AS remains to be resolved, however, based on their roles in humans and our research, future research will likely implicate their involvement numerous abiotic stresses through the regulation of AS.

## Materials and methods

### Phylogenetic trees

Amino acid sequences of the protein families were acquired from TAIR (https://www.arabidopsis.org/) (SRPK1; AT4G35500, SRPK2; AT2G17530, SRPK3; AT5G22840, SRPK4; AT3G53030, SRPK5; AT3G44850, AFC1; AT3G53570, AFC2; AT4G24740, AFC3; AT4G32660, PRP4Ka; AT3G25840, PRP4Kb; AT1G13350, PRP4Kc; AT3G53640). Amino acid sequences were aligned using MAFFT, version 7 (http://mafft.cbrc.jp/alignment/server/; [134]) and input into HMMER3 v3.3.2 (http://hmmer.org/) to acquire a protein profile. Organism proteomes were acquired from Phytozome (https://phytozome.jgi.doe.gov/pz/portal.html). Protein family profiles were used to query against full proteomes using HMMER3. For organisms whose proteome is not available, their orthologs were acquired by using the 1KP database [135] using BLASTp (https://db.cngb.org/blast/blast/blastp/?project=onekp). Organisms whose orthologs were acquired using full proteomes or using the 1KP project (https://sites.google.com/a/ualberta.ca/onekp/; Supplemental Table 1). Hit above natural e-value cut off range were select for reciprocal BLAST against Arabidopsis proteome to ascertain orthology. Compiled sequences were aligned using MAFFT E-NS-I for SRPK and MAFFT L-INS-I for AFC and PRP4K. Alignment was inspected using TCS (http://tcoffee.crg.cat/apps/tcoffee/do:core) and manually trimmed using GeneDoc (insert software citation) to remove gaps containing higher than 90 % gaps between sequences (Supplemental File 1, 2, and 3). The first maximum likelihood tree was generated using IQtree ([136] http://iqtree.cibiv.univie.ac.at/) using 1000 bootstrap alignments, 1000 iterations, and 0.99 minimum correlation coefficient parameters. The Baseyian tree was generated using Mr.Bayes with CIPRES ([137] https://www.phylo.org/) with the following parameter: 50 000 000 ngen MCMC, nruns = 2, nchains = 4. Maximum likelihood = 10,000 bootstraps, 1000 iterations, min r2 = 0.99 (Supplemental File 5). The second maximum likelihood tree was generated using PhyML version 3.0 (Guindon et al., 2011; http://www.atgc-montpellier.fr/phyml/, [138]) with LG amino acid substitution model, aLRT SH-like fast likelihood-based method for branch support, and all other parameters set as default.

### Domain conservation analysis

The putative orthologous amino acid sequences that were found using HMMER (http://hmmer.org/) and 1kp project were compiled for domain analysis (Supplemental Table 1). Compiled peptide sequences were separated into the following phylogenetic groups: opisthokonts, SAR (stramenopiles, alveolates, rhizaria), hacrobia, rhodophytes, chlorophytes, bryophytes, gymnosperms, monocots, and eudicots. Each phylogenetic group was inputted into DomainViz ([57] https://uhrigprotools.biology.ualberta.ca/domainviz) with the following settings: minimum domain prevalence (0.05) and minimum domain position conservation (0.05).

### Percent similarity of HsSRPKs and AtSRPKs protein sequences

Human and Arabidopsis SRPK compiled sequences were aligned using MAFFT LINS-I with default settings. Resulting alignment was inputted through Sequence Identities And Similarities (SIAS) tool (http://imed.med.ucm.es/Tools/sias.html) with BLOSUM 62 as the scoring matrix. The length of the MSA was used as the sequence length denominator used in the percent identity equation.

### Cis-regulatory elements data search

Promoter elements present in the sequence of the investigated splicing-related kinases were searched using AtCisDB. Arabidopsis gene ID (AGI) of each splicing-related kinase was input into the AtCisDB database ([68] https://agris-knowledgebase.org/AtcisDB/).

### Developmental, abiotic, and photoperiod transcript expression analysis

Relative expression levels of slicing kinases were acquired with Hierarchical Clustering tool from GENEVESTIGATOR database (https://genevestigator.com/; [83]). BAR Arabidopsis eFP browser database for extracting microarray transcript expression data (https://bar.utoronto.ca/efp/cgi-bin/efpWeb.cgi; [94]) was mined for developmental and abiotic stress transcriptional changes. Absolute values, which are the raw values provided by BAR, were log_2_ transformed. Abiotic stress values were normalized against the control. Photoperiod transcript changes were mined using DiurnalDb [http://diurnal.mocklerlab.org/diurnal_data_finders/new; [123]).

### Plant growth

Sterilized *A. thaliana* wild type *Col-0* seeds (source ABRC; abrc.osu.edu/) were plated on 0.5 × MS (Caisson Laboratories inc. Murashige & Skoog MSP01-50LT) 0.8 % plant agar (Caisson laboratories inc. Phytoblend™ PTP01-2 KG) and stratified for 3 days at 4 °C in the dark (Supplemental Figure 7). Seeds were germinated and grow for 7 days under 8 h: 16 h, 12 h: 12 h or 16 h: 8 h photoperiod. Seedlings were grown vertically in custom 3D printed blacked-out vertical plate holders to minimize root exposure to light. For 24 h: 0 h photoperiod, seedlings were transferred from 12 h: 12 h after 5 days of entrainment to 24 h: 0 h for 2 days. Plant tissue was harvested at ZT6, ZT11, ZT18 and ZT23. All seedlings were grown under LED light with 100 µmol/m^2^/s at constant 22 ^o^C with 50 % humidity.

### Abiotic stress experimentation

All abiotic stress seedlings were grown in 12 h: 12 h photoperiod. Cold treated seedlings were placed in 4 °C thermo-regulated vertical plate holder at ZT 9 on day 6, then harvested at ZT9 on day 7. Heat treated seedlings were transferred at ZT1 on day 7 to a 38 °C thermo-regulated vertical place holder for 3 h, then placed back at 22 °C for a 3 h recovery time at which point seedlings were harvested at ZT7. Osmotic and salt stressed seedling were carefully transferred at ZT6 from germination media to 300 mM mannitol and 150 mM NaCl to simulate osmotic and salt stress, respectively. Seedlings were exposed for 24 h and harvested the subsequent day at ZT6. All seedlings were grown under LED light with 100 µmol/m^2^/s at constant 22 °C with 50 % humidity (Supplemental Figure 7).

### Tissue processing for transcript quantification

Four biological replicates each containing ~ 100 mg of plant tissue were flash frozen and ground using Geno/Grinder® for 30 s at 1200 rpm. Total RNA was extracted using a modified TRizol protocol [139]. 1 ml of TRI Reagent® (Sigma-Aldrich T9424) was added to each 100 mg of tissue and incubated for 10 min at room temperature. Extracellular material was removed by centrifuging at 13,000 × g for 10 min. Supernatant was transferred to new tubes and 200 µL of chloroform was added. Tubes were then inverted variously for 15 s and then left at RT for 3 min. Phase separation was achieved by centrifuging at 13,000 × g for 15 min at 4 °C. The aqueous phase was transferred to new tubes carefully avoiding the interphase. RNA was precipitated by adding 500 µL of 100 % isopropanol and then left to incubate for 10 min at RT. RNA was pelleted by centrifuging at 13,000 × g for 15 min at 4 °C. Supernatant was removed and pellet was washed with 1 ml of 75% Ethanol. Tube were centrifuged once again at 7500 × g for 5 min at 4 °C. Pellet was dried and subsequently resuspended in nuclease-free water. Total RNA was quantified by NanoDrop ND 1000 spectrophotometer. A minimum of 150 ng of purified RNA in 7.5 µL was sent to NanoString (NanoString Technologies, Seattle, USA, https://www.nanostring.com) for analysis. The NanoString probes were designed and synthesized by NanoString (Supplemental Table 8). PP2AA3 (AT1G13320) and UBQ10 (AT4G05320) were used as reference genes (Czechowski et al., 2005; Hong et al., 2010). All data was normalized the corresponding abundance of PP2AA3 for each sample, with Log2 fold change (FC) calculated between control and stress conditions, with the corrected *p*-value (FDR) calculated using Benjamini-Yekutieli. PP2AA3 (AT1G13320) and UBQ10 (AT4G05320) were used as reference genes [140, 141]. ZT6 12 h: 12 h tissue was used as a control comparison during data analysis. Positive control genes were selected for each abiotic stress and photoperiod. COR78 (AT5G52310) for cold, HOP3 (AT4G12400) for heat, LEA4-5 (AT5G06760) for osmotic, salt and cold, and KIN1 (AT5G15960) for osmotic, salt, and cold (Supplemental Figure 8). CCA1 (AT2G46830) and TOC1 (AT5G61380) were used for photoperiod (Supplemental Figure 9).

## Supplementary Information

**Additional file 1.**

**Additional file 2.**

**Additional file 3.**

**Additional file 4.**

**Additional file 5.**

**Additional file 6.**

**Additional file 7.**

**Additional file 8.**

**Additional file 9.**

**Additional file 10.**

**Additional file 11.**

**Additional file 12.**

**Additional file 13.**

**Additional file 14.**

**Additional file 15.**

**Additional file 16.**

**Additional file 17.**

**Additional file 18.**

**Additional file 19.**

**Additional file 20.**

**Additional file 21.**

**Additional file 22.**

**Additional file 23.**

## Data Availability

All data generated or analysed during this study are included in this published article and its supplementary information files.
