# Correction: Genome-scale analysis of *Arabidopsis* splicing-related protein kinase families reveals roles in abiotic stress adaptation

**DOI:** 10.1186/s12870-023-04289-6

**Published:** 2023-05-23

**Authors:** M. C. Rodriguez Gallo, Q. Li, D. Mehta, R. G. Uhrig

**Affiliations:** 1 Department of Biological Sciences, University of Alberta, Edmonton, AB T6G 2E9 Canada; 2 Department of Biochemistry, University of Alberta, Edmonton, AB Canada

**Correction:** ***BMC Plant Biol*** **22, 496 (2022)**

10.1186/s12870-022-03870-9

Following publication of the original article [1], the authors identified an error in the author name of D. Mehta. The author name was not captured correctly.

The correct author name should be:

Given name: D

Family name: Mehta

The author group has been updated above and the original article [1] has been corrected.
