# Supplementary material for: Genome-scale analysis of Arabidopsis splicing-related protein kinase families reveals roles in abiotic stress adaptation
Source: BMC Plant Biol. 2022 Oct 22;22:496. doi: 10.1186/s12870-022-03870-9 (PMC9587599; doi:10.1186/s12870-022-03870-9)
Supplement: Supplementary file 1 — Additional file 1. [file 12870_2022_3870_MOESM1_ESM.pdf]

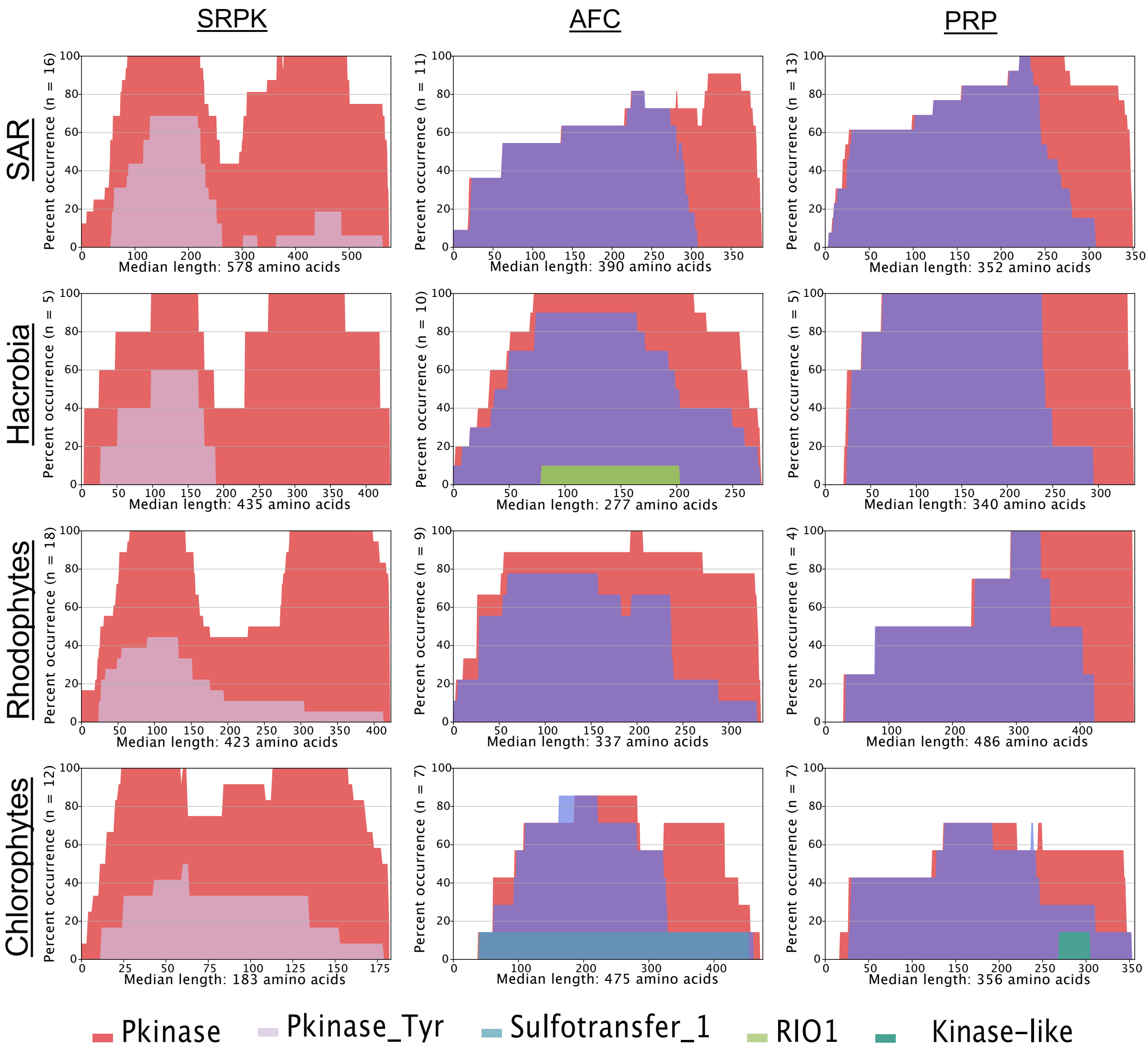

SRPK
AFC
PRP
SAR
Hacrobia
Rhodophytes
Chlorophytes
Percent occurrence (n = 16)
Percent occurrence (n = 11)
Percent occurrence (n = 13)
Percent occurrence (n = 5)
Percent occurrence (n = 10)
Percent occurrence (n = 5)
Percent occurrence (n = 18)
Percent occurrence (n = 9)
Percent occurrence (n = 4)
Percent occurrence (n = 12)
Percent occurrence (n = 7)
Percent occurrence (n = 7)
Median length: 578 amino acids
Median length: 390 amino acids
Median length: 352 amino acids
Median length: 435 amino acids
Median length: 277 amino acids
Median length: 340 amino acids
Median length: 423 amino acids
Median length: 337 amino acids
Median length: 486 amino acids
Median length: 183 amino acids
Median length: 475 amino acids
Median length: 356 amino acids
Pkinase
Pkinase_Tyr
Sulfotransfer_1
RIO1
Kinase-like
