# Supplementary material for: Genome-scale analysis of Arabidopsis splicing-related protein kinase families reveals roles in abiotic stress adaptation
Source: BMC Plant Biol. 2022 Oct 22;22:496. doi: 10.1186/s12870-022-03870-9 (PMC9587599; doi:10.1186/s12870-022-03870-9)
Supplement: Supplementary file 5 — Additional file 5. [file 12870_2022_3870_MOESM5_ESM.pdf]

Supplemental File 4

The follow file contains raw phylogenetic tress for SRPK, AFC, and PRP4K protein families. Each tree contains node supports and is rooted to human orthologous proteins.

the order of the trees is as follows:
- maximum likelihood (IQTree) SRPK
- maximum likelihood (IQTree) AFC
- maximum likelihood (IQTree) PRP4K
- Bayesian (Mr.Bayes) SRPK
- Bayesian (Mr.Bayes) AFC
- Bayesian (Mr.Bayes) PRP4K
- maximum likelihood (PhyML) SRPK
- maximum likelihood (PhyML) AFC
- maximum likelihood (PhyML) PRP4K

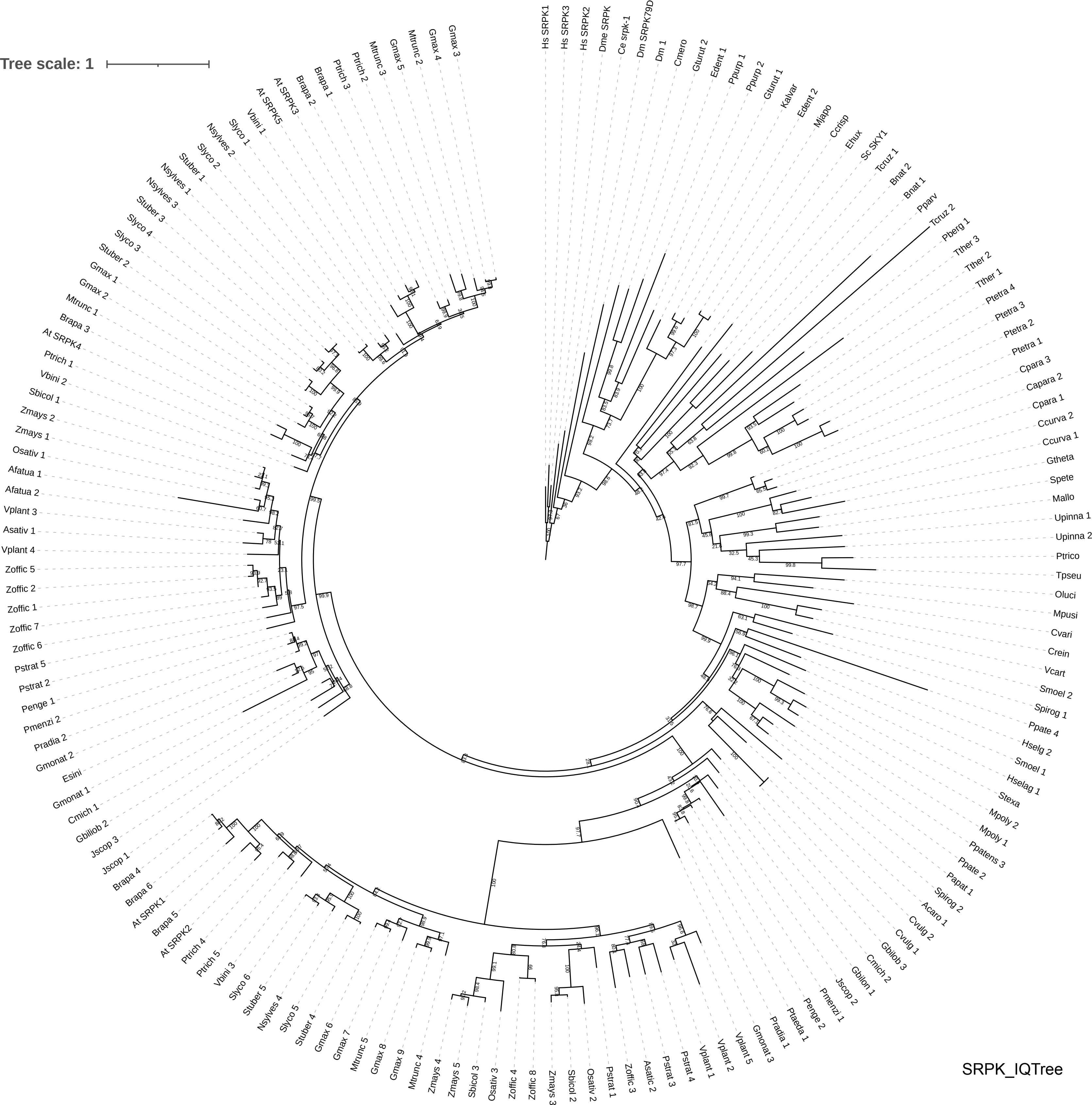
Tree scale: 1
Hs SRPK1
Hs SRPK3
Hs SRPK2
Dme SRPK
Ce srpk-1
Dm SRPK79D
Dm 1
Cmero
Gturut 2
Edent 1
Ppurp 1
Ppurp 2
Gturut 1
Kalvar
Edent 2
Mjapo
Ccrisp
Ehux
Sc SKY1
Tcruz 1
Bnat 2
Bnat 1
Pparv
Tcruz 2
Pberg 1
Tther 3
Tther 2
Tther 1
Ptetra 4
Ptetra 3
Ptetra 2
Ptetra 1
Cpara 3
Capara 2
Cpara 1
Ccurva 2
Ccurva 1
Gtheta
Spete
Mallo
Upinna 1
Upinna 2
Ptrico
Tpseu
Oluci
Mpusi
Cvari
Crein
Vcart
Smoel 2
Spirog 1
Ppate 4
Hselg 2
Smoel 1
Hselag 1
Stexa
Mpoly 2
Mpoly 1
Ppatens 3
Ppate 2
Papat 1
Spirog 2
Acaro 1
Cvulg 2
Cvulg 1
Gbiliob 3
Cmich 2
Gbilon 1
Jscop 2
Pmenzi 1
Penge 2
Ptaeda 1
Pradia 1
Gmonat 3
Vplant 5
Vplant 2
Vplant 1
Pstrat 4
Pstrat 3
Asatic 2
Zoffic 3
Pstrat 1
Osativ 2
Sbicol 2
Zmays 3
Zoffic 8
Zoffic 4
Osativ 3
Sbicol 3
Zmays 5
Zmays 4
Mtrunc 4
Gmax 9
Gmax 8
Mtrunc 5
Gmax 7
Gmax 6
Stuber 4
Slyco 5
Nsylves 4
Stuber 5
Slyco 6
Vbini 3
Ptrich 5
Ptrich 4
At SRPK2
Brapa 5
At SRPK1
Brapa 6
Brapa 4
Jscop 1
Jscop 3
Gbiliob 2
Cmich 1
Gmonat 1
Esini
Gmonat 2
Pradia 2
Pmenzi 2
Penge 1
Pstrat 2
Pstrat 5
Zoffic 6
Zoffic 7
Zoffic 1
Zoffic 2
Zoffic 5
Vplant 4
Asativ 1
Vplant 3
Afatua 2
Afatua 1
Osativ 1
Zmays 1
Zmays 2
Sbicol 1
Vbini 2
Ptrich 1
At SRPK4
Brapa 3
Mtrunc 1
Gmax 2
Gmax 1
Stuber 2
Slyco 3
Slyco 4
Stuber 3
Nsylves 3
Nsylves 1
Stuber 1
Slyco 2
Nsylves 2
Slyco 1
Vbini 1
At SRPK5
At SRPK3
Brapa 2
Brapa 1
Ptrich 3
Ptrich 2
Mtrunc 3
Gmax 5
Mtrunc 2
Gmax 4
Gmax 3
SRPK_IQTree

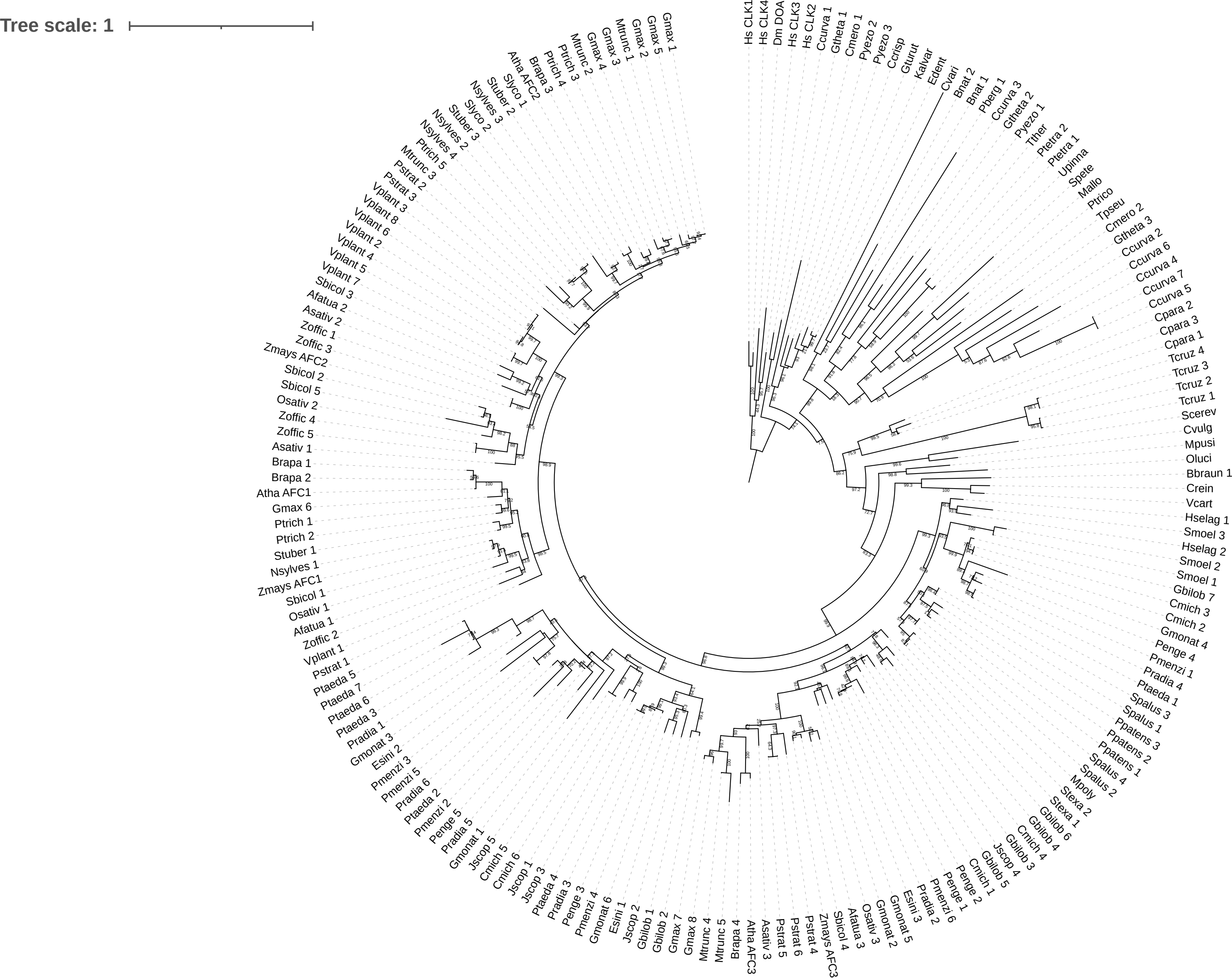
Tree scale: 1
Hs CLK1
Hs CLK4
Dm DOA
Hs CLK3
Hs CLK2
Ccurva 1
Gtheta 1
Cmero 1
Pyezo 2
Pyezo 3
Ccrisp
Gturut
Kalvar
Edent
Cvari
Bnat 2
Bnat 1
Pberg 1
Ccurva 3
Gtheta 2
Pyezo 1
Tther
Ptetra 2
Ptetra 1
Upinna
Spete
Mallo
Ptrico
Tpseu
Cmero 2
Gtheta 3
Ccurva 2
Ccurva 6
Ccurva 4
Ccurva 7
Ccurva 5
Cpara 2
Cpara 3
Cpara 1
Tcruz 4
Tcruz 3
Tcruz 2
Tcruz 1
Scerev
Cvulg
Mpusi
Oluci
Bbraun 1
Crein
Vcart
Hselag 1
Smoel 3
Hselag 2
Smoel 2
Smoel 1
Gbilob 7
Cmich 3
Cmich 2
Gmonat 4
Penge 4
Pmenzi 1
Pradia 4
Ptaeda 1
Spalus 3
Spalus 1
Ppatens 3
Ppatens 2
Ppatens 1
Spalus 4
Spalus 2
Mpoly
Stexa 2
Stexa 1
Gbilob 6
Gbilob 4
Cmich 4
Gbilob 3
Jscop 4
Gbilob 5
Cmich 1
Penge 2
Penge 1
Pmenzi 6
Pradia 2
Esini 3
Gmonat 5
Gmonat 2
Osativ 3
Afatua 3
Sbicol 4
Zmays AFC3
Pstrat 4
Pstrat 6
Pstrat 5
Asativ 3
Atha AFC3
Brapa 4
Mtrunc 5
Mtrunc 4
Gmax 8
Gmax 7
Gbilob 2
Gbilob 1
Jscop 2
Esini 1
Gmonat 6
Pmenzi 4
Penge 3
Pradia 3
Ptaeda 4
Jscop 3
Jscop 1
Cmich 6
Cmich 5
Jscop 5
Gmonat 1
Pradia 5
Penge 5
Pmenzi 2
Ptaeda 2
Pradia 6
Pmenzi 5
Pmenzi 3
Esini 2
Gmonat 3
Pradia 1
Ptaeda 3
Ptaeda 6
Ptaeda 7
Ptaeda 5
Pstrat 1
Vplant 1
Zoffic 2
Afatua 1
Osativ 1
Sbicol 1
Zmays AFC1
Nsylves 1
Stuber 1
Ptrich 2
Ptrich 1
Gmax 6
Atha AFC1
Brapa 2
Brapa 1
Asativ 1
Zoffic 5
Zoffic 4
Osativ 2
Sbicol 5
Sbicol 2
Zmays AFC2
Zoffic 3
Zoffic 1
Asativ 2
Afatua 2
Sbicol 3
Vplant 7
Vplant 5
Vplant 4
Vplant 2
Vplant 6
Vplant 8
Vplant 3
Pstrat 3
Pstrat 2
Mtrunc 3
Ptrich 5
Nsylves 4
Nsylves 2
Stuber 3
Slyco 2
Nsylves 3
Stuber 2
Slyco 1
Atha AFC2
Brapa 3
Ptrich 4
Ptrich 3
Mtrunc 2
Gmax 4
Gmax 3
Mtrunc 1
Gmax 2
Gmax 5
Gmax 1

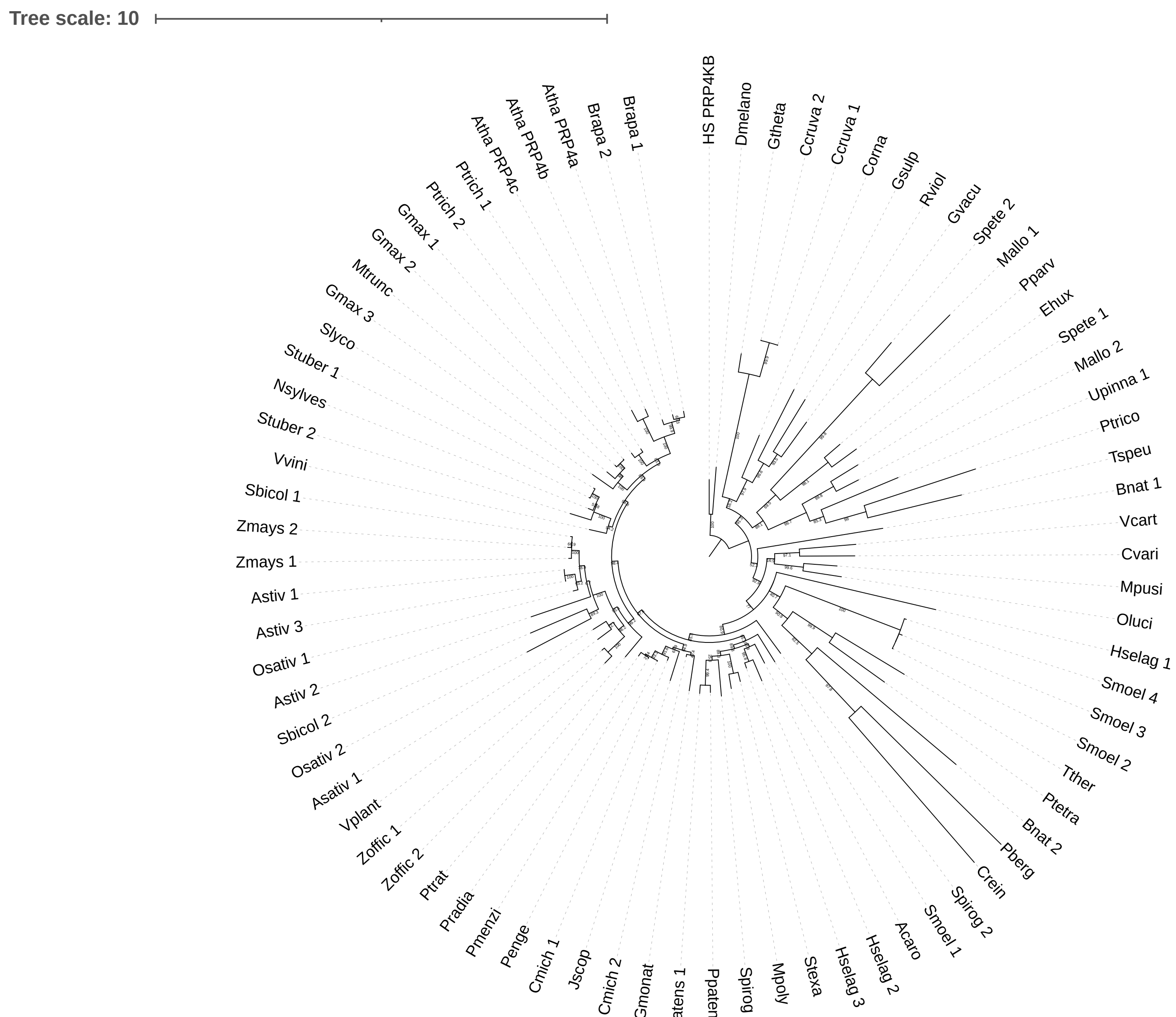

Tree scale: 10
HS PRP4KB
Dmelano
Gtheta
Ccruva 2
Ccruva 1
Corna
Gsulp
Rviol
Gvacu
Spete 2
Mallo 1
Pparv
Ehux
Spete 1
Mallo 2
Upinna 1
Ptrico
Tspeu
Bnat 1
Vcart
Cvari
Mpusi
Oluci
Hselag 1
Smoel 4
Smoel 3
Smoel 2
Tther
Ptetra
Bnat 2
Pberg
Crein
Spirog 2
Smoel 1
Acaro
Hselag 2
Hselag 3
Stexa
Mpoly
Spirog
Gmonat
Cmich 2
Jscop
Cmich 1
Penge
Pmenzi
Pradia
Ptrat
Zoffic 2
Zoffic 1
Vplant
Asativ 1
Osativ 2
Sbicol 2
Astiv 2
Osativ 1
Astiv 3
Astiv 1
Zmays 1
Zmays 2
Sbicol 1
Vvini
Stuber 2
Nsylves
Stuber 1
Slyco
Gmax 3
Mtrunc
Gmax 2
Gmax 1
Ptrich 2
Ptrich 1
Atha PRP4c
Atha PRP4b
Atha PRP4a
Brapa 2
Brapa 1

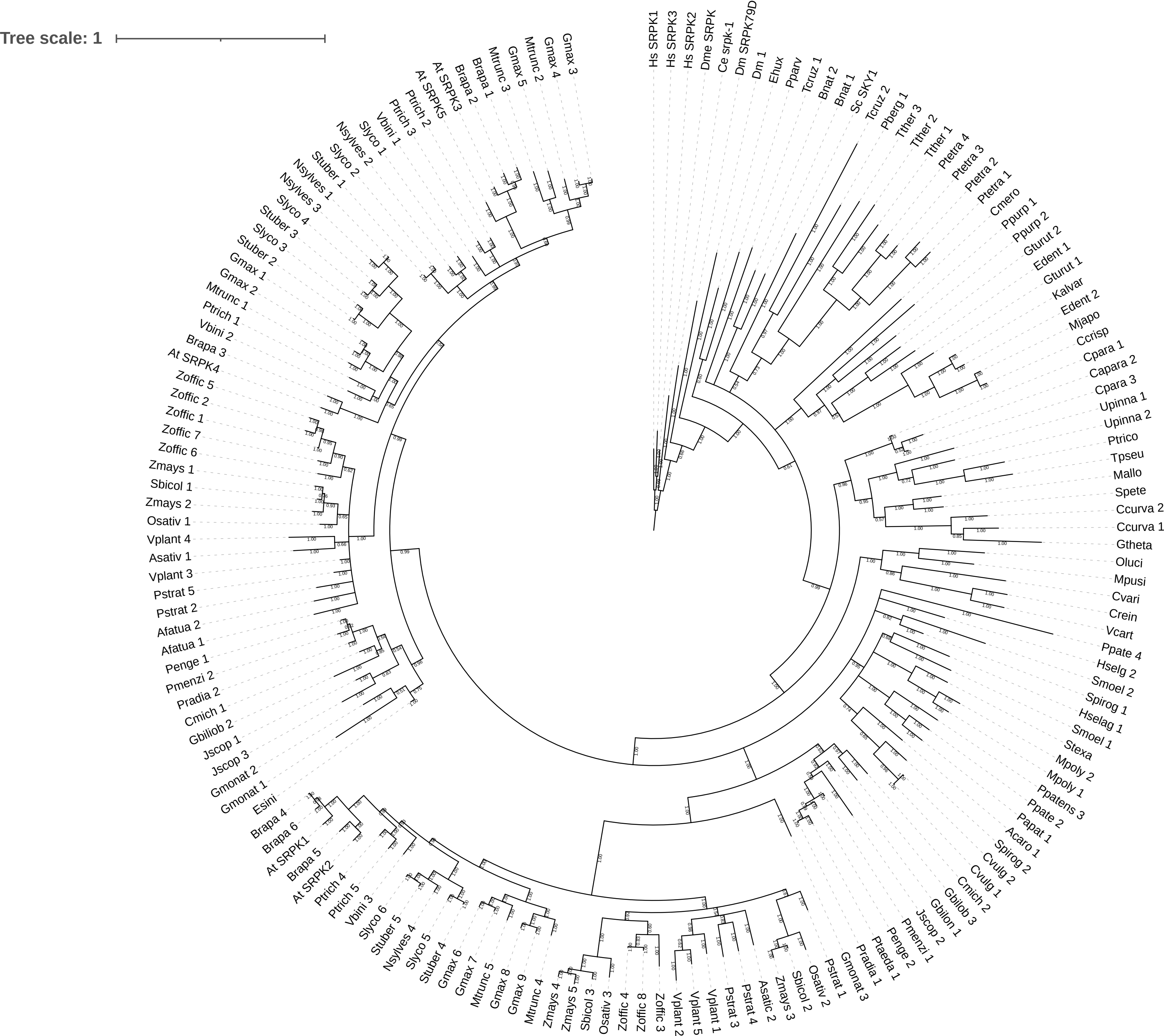
Tree scale: 1
Hs SRPK1
Hs SRPK3
Hs SRPK2
Dme SRPK
Ce srpk-1
Dm SRPK79D
Dm 1
Ehux
Pparv
Tcruz 1
Bnat 2
Bnat 1
Sc SKY1
Tcruz 2
Pberg 1
Tther 3
Tther 2
Tther 1
Ptetra 4
Ptetra 3
Ptetra 2
Ptetra 1
Cmero
Ppurp 1
Ppurp 2
Gturut 2
Edent 1
Gturut 1
Kalvar
Edent 2
Mjapo
Ccrisp
Cpara 1
Capara 2
Cpara 3
Upinna 1
Upinna 2
Ptrico
Tpseu
Mallo
Spete
Ccurva 2
Ccurva 1
Gtheta
Oluci
Mpusi
Cvari
Crein
Vcart
Ppate 4
Hselg 2
Smoel 2
Spirog 1
Hselag 1
Smoel 1
Stexa
Mpoly 2
Mpoly 1
Ppatens 3
Ppate 2
Papat 1
Acaro 1
Spirog 2
Cvulg 2
Cvulg 1
Cmich 2
Gbilob 3
Gbilon 1
Jscop 2
Pmenzi 1
Penge 2
Ptaeda 1
Pradia 1
Gmonat 3
Pstrat 1
Osativ 2
Sbicol 2
Zmays 3
Asatic 2
Pstrat 4
Pstrat 3
Vplant 1
Vplant 5
Vplant 2
Zoffic 3
Zoffic 8
Zoffic 4
Osativ 3
Sbicol 3
Zmays 5
Zmays 4
Mtrunc 4
Gmax 9
Gmax 8
Mtrunc 5
Gmax 7
Gmax 6
Stuber 4
Slyco 5
Nsylves 4
Stuber 5
Slyco 6
Vbini 3
Ptrich 5
Ptrich 4
At SRPK2
Brapa 5
At SRPK1
Brapa 6
Brapa 4
Esini
Gmonat 1
Gmonat 2
Jscop 3
Jscop 1
Gbiliob 2
Cmich 1
Pradia 2
Pmenzi 2
Penge 1
Afatua 1
Afatua 2
Pstrat 2
Pstrat 5
Vplant 3
Asativ 1
Vplant 4
Osativ 1
Zmays 2
Sbicol 1
Zmays 1
Zoffic 6
Zoffic 7
Zoffic 1
Zoffic 2
Zoffic 5
At SRPK4
Brapa 3
Vbini 2
Ptrich 1
Mtrunc 1
Gmax 2
Gmax 1
Stuber 2
Slyco 3
Stuber 3
Slyco 4
Nsylves 3
Nsylves 1
Stuber 1
Slyco 2
Nsylves 2
Slyco 1
Vbini 1
Ptrich 3
Ptrich 2
At SRPK5
At SRPK3
Brapa 2
Brapa 1
Mtrunc 3
Gmax 5
Mtrunc 2
Gmax 4
Gmax 3

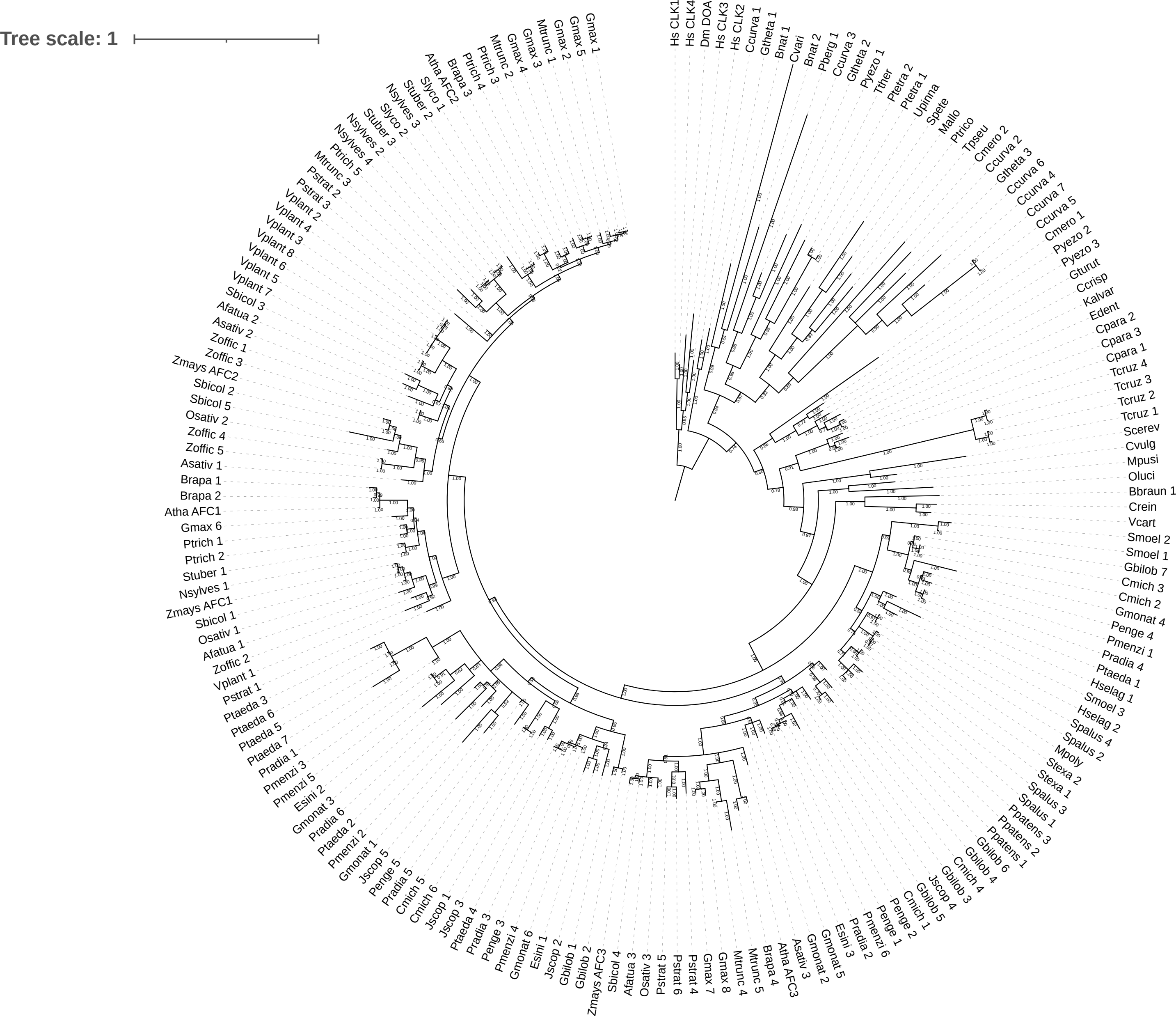

Tree scale: 1
Hs CLK1
Hs CLK4
Dm DOA
Hs CLK3
Hs CLK2
Ccurva 1
Gtheta 1
Bnat 1
Cvari
Bnat 2
Pberg 1
Ccurva 3
Gtheta 2
Pyezo 1
Tther
Ptetra 2
Ptetra 1
Upinna
Spete
Mallo
Ptrico
Tpseu
Cmero 2
Ccurva 2
Gtheta 3
Ccurva 6
Ccurva 4
Ccurva 7
Ccurva 5
Cmero 1
Pyezo 2
Pyezo 3
Gturut
Ccrisp
Kalvar
Edent
Cpara 2
Cpara 3
Cpara 1
Tcruz 4
Tcruz 3
Tcruz 2
Tcruz 1
Scerev
Cvulg
Mpusi
Oluci
Bbraun 1
Crein
Vcart
Smoel 2
Smoel 1
Gbilob 7
Cmich 3
Cmich 2
Gmonat 4
Penge 4
Pmenzi 1
Pradia 4
Ptaeda 1
Hselag 1
Smoel 3
Hselag 2
Spalus 4
Spalus 2
Mpoly
Stexa 2
Stexa 1
Spalus 3
Spalus 1
Ppatens 3
Ppatens 2
Ppatens 1
Gbilob 6
Gbilob 4
Cmich 4
Gbilob 3
Jscop 4
Gbilob 5
Cmich 1
Penge 2
Penge 1
Pmenzi 6
Pradia 2
Esini 3
Gmonat 5
Gmonat 2
Asativ 3
Atha AFC3
Brapa 4
Mtrunc 5
Mtrunc 4
Gmax 8
Gmax 7
Pstrat 4
Pstrat 6
Pstrat 5
Osativ 3
Afatua 3
Sbicol 4
Zmays AFC3
Gbilob 2
Gbilob 1
Jscop 2
Esini 1
Gmonat 6
Pmenzi 4
Penge 3
Pradia 3
Ptaeda 4
Jscop 3
Jscop 1
Cmich 6
Cmich 5
Pradia 5
Penge 5
Jscop 5
Gmonat 1
Pmenzi 2
Ptaeda 2
Pradia 6
Gmonat 3
Esini 2
Pmenzi 5
Pmenzi 3
Pradia 1
Ptaeda 7
Ptaeda 5
Ptaeda 6
Ptaeda 3
Pstrat 1
Vplant 1
Zoffic 2
Afatua 1
Osativ 1
Sbicol 1
Zmays AFC1
Nsylves 1
Stuber 1
Ptrich 2
Ptrich 1
Gmax 6
Atha AFC1
Brapa 2
Brapa 1
Asativ 1
Zoffic 5
Zoffic 4
Osativ 2
Sbicol 5
Sbicol 2
Zmays AFC2
Zoffic 3
Zoffic 1
Asativ 2
Afatua 2
Sbicol 3
Vplant 7
Vplant 5
Vplant 6
Vplant 8
Vplant 3
Vplant 4
Vplant 2
Pstrat 3
Pstrat 2
Mtrunc 3
Ptrich 5
Nsylves 4
Nsylves 2
Stuber 3
Slyco 2
Nsylves 3
Stuber 2
Slyco 1
Atha AFC2
Brapa 3
Ptrich 4
Ptrich 3
Mtrunc 2
Gmax 4
Gmax 3
Mtrunc 1
Gmax 2
Gmax 5
Gmax 1

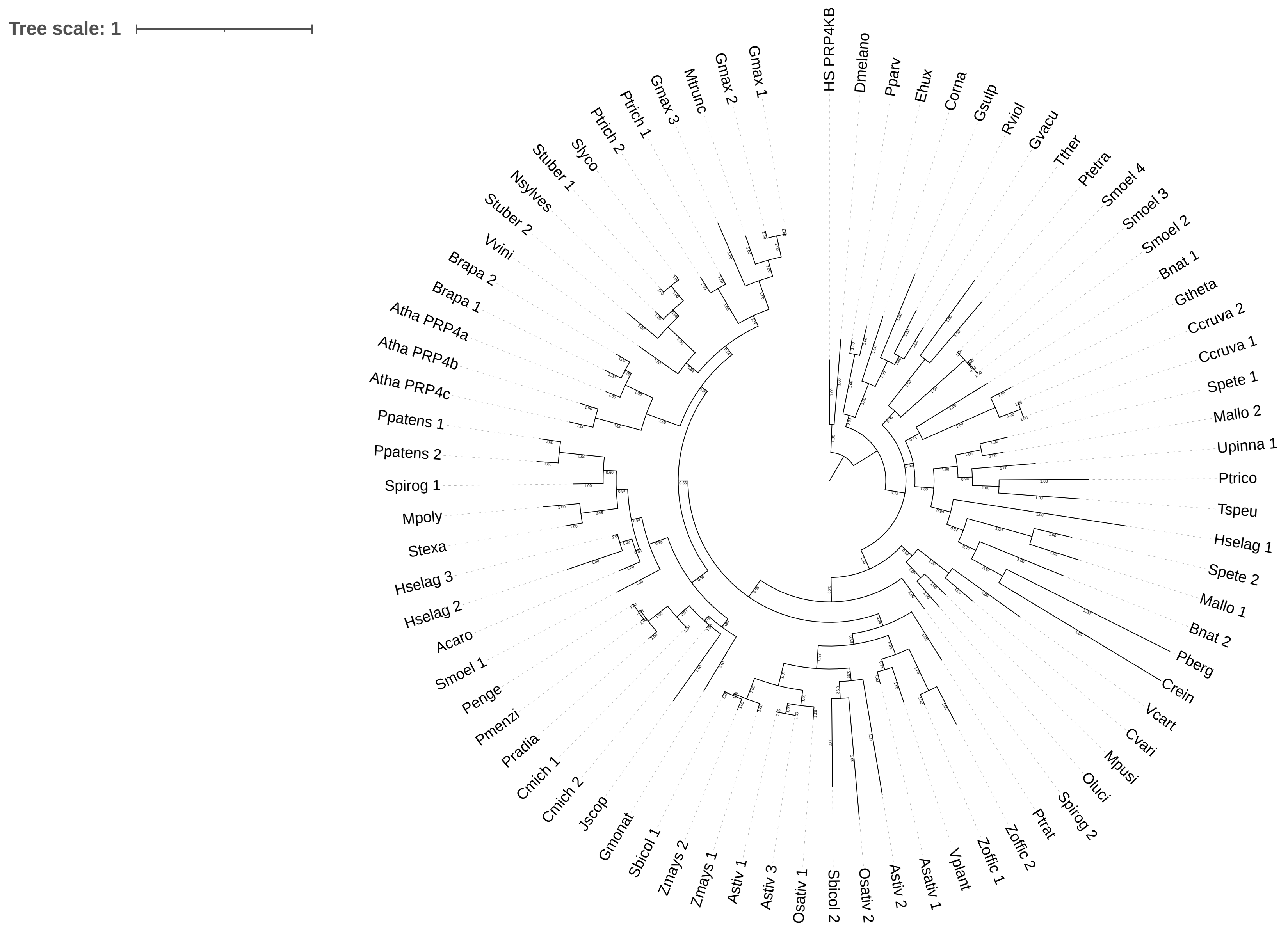

Tree scale: 1
HS PRP4KB
Dmelano
Pparv
Ehux
Corna
Gsulp
Rviol
Gvacu
Tther
Ptetra
Smoel 4
Smoel 3
Smoel 2
Bnat 1
Gtheta
Ccruva 2
Ccruva 1
Spete 1
Mallo 2
Upinna 1
Ptrico
Tspeu
Hselag 1
Spete 2
Mallo 1
Bnat 2
Pberg
Crein
Vcart
Cvari
Mpusi
Oluci
Spirog 2
Ptrat
Zoffic 2
Zoffic 1
Vplant
Asativ 1
Astiv 2
Osativ 2
Sbicol 2
Osativ 1
Astiv 3
Astiv 1
Zmays 1
Zmays 2
Sbicol 1
Gmonat
Jscop
Cmich 2
Cmich 1
Pradia
Pmenzi
Penge
Smoel 1
Acaro
Hselag 2
Hselag 3
Stexa
Mpoly
Spirog 1
Ppatens 2
Ppatens 1
Atha PRP4c
Atha PRP4b
Atha PRP4a
Brapa 1
Brapa 2
Vvini
Stuber 2
Nsylves
Stuber 1
Slyco
Ptrich 2
Ptrich 1
Gmax 3
Mtrunc
Gmax 2
Gmax 1

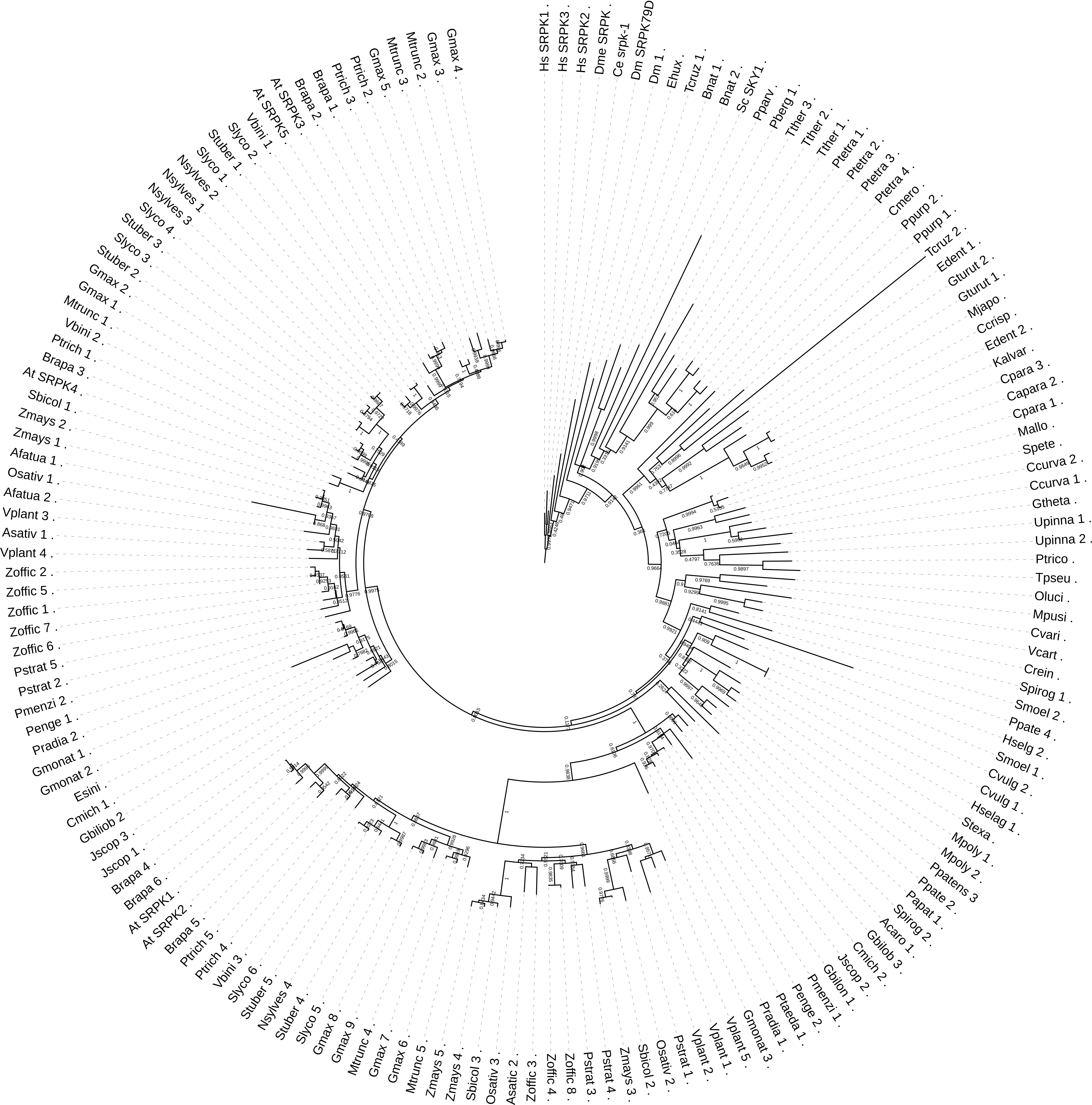

Hs SRPK1
Hs SRPK3
Hs SRPK2
Dme SRPK
Ce srpk-1
Dm SRPK79D
Dm 1
Ehux
Tcruz 1
Bnat 1
Bnat 2
Sc SKY1
Pparv
Pberg 1
Tther 3
Tther 2
Tther 1
Ptetra 1
Ptetra 2
Ptetra 3
Ptetra 4
Cmero
Ppurp 2
Ppurp 1
Tcruz 2
Edent 1
Gturut 2
Gturut 1
Mjapo
Ccrisp
Edent 2
Kalvar
Cpara 3
Capara 2
Cpara 1
Mallo
Spete
Ccurva 2
Ccurva 1
Gtheta
Upinna 1
Upinna 2
Ptrico
Tpseu
Oluci
Mpusi
Cvari
Vcart
Crein
Spirog 1
Smoel 2
Ppate 4
Hselg 2
Smoel 1
Cvulg 2
Cvulg 1
Hselag 1
Stexa
Mpoly 1
Mpoly 2
Ppatens 3
Ppate 2
Papat 1
Spirog 2
Acaro 1
Gbiliob 3
Cmich 2
Jscop 2
Gbilon 1
Pmenzi 1
Penge 2
Ptaeda 1
Pradia 1
Gmonat 3
Vplant 5
Vplant 1
Vplant 2
Pstrat 1
Osativ 2
Sbicol 2
Zmays 3
Pstrat 4
Pstrat 3
Zoffic 8
Zoffic 4
Zoffic 3
Asatic 2
Osativ 3
Sbicol 3
Zmays 4
Zmays 5
Mtrunc 5
Gmax 6
Gmax 7
Mtrunc 4
Gmax 9
Gmax 8
Slyco 5
Stuber 4
Nsylves 4
Stuber 5
Slyco 6
Vbini 3
Ptrich 4
Ptrich 5
Brapa 5
At SRPK2
At SRPK1
Brapa 6
Brapa 4
Jscop 1
Jscop 3
Gbiliob 2
Cmich 1
Esini
Gmonat 2
Gmonat 1
Pradia 2
Penge 1
Pmenzi 2
Pstrat 2
Pstrat 5
Zoffic 6
Zoffic 7
Zoffic 1
Zoffic 5
Zoffic 2
Vplant 4
Asativ 1
Vplant 3
Afatua 2
Osativ 1
Afatua 1
Zmays 1
Zmays 2
Sbicol 1
At SRPK4
Brapa 3
Ptrich 1
Vbini 2
Mtrunc 1
Gmax 1
Gmax 2
Stuber 2
Slyco 3
Stuber 3
Slyco 4
Nsylves 3
Nsylves 1
Nsylves 2
Slyco 1
Stuber 1
Slyco 2
Vbini 1
At SRPK5
At SRPK3
Brapa 2
Brapa 1
Ptrich 3
Ptrich 2
Gmax 5
Mtrunc 3
Mtrunc 2
Gmax 3
Gmax 4

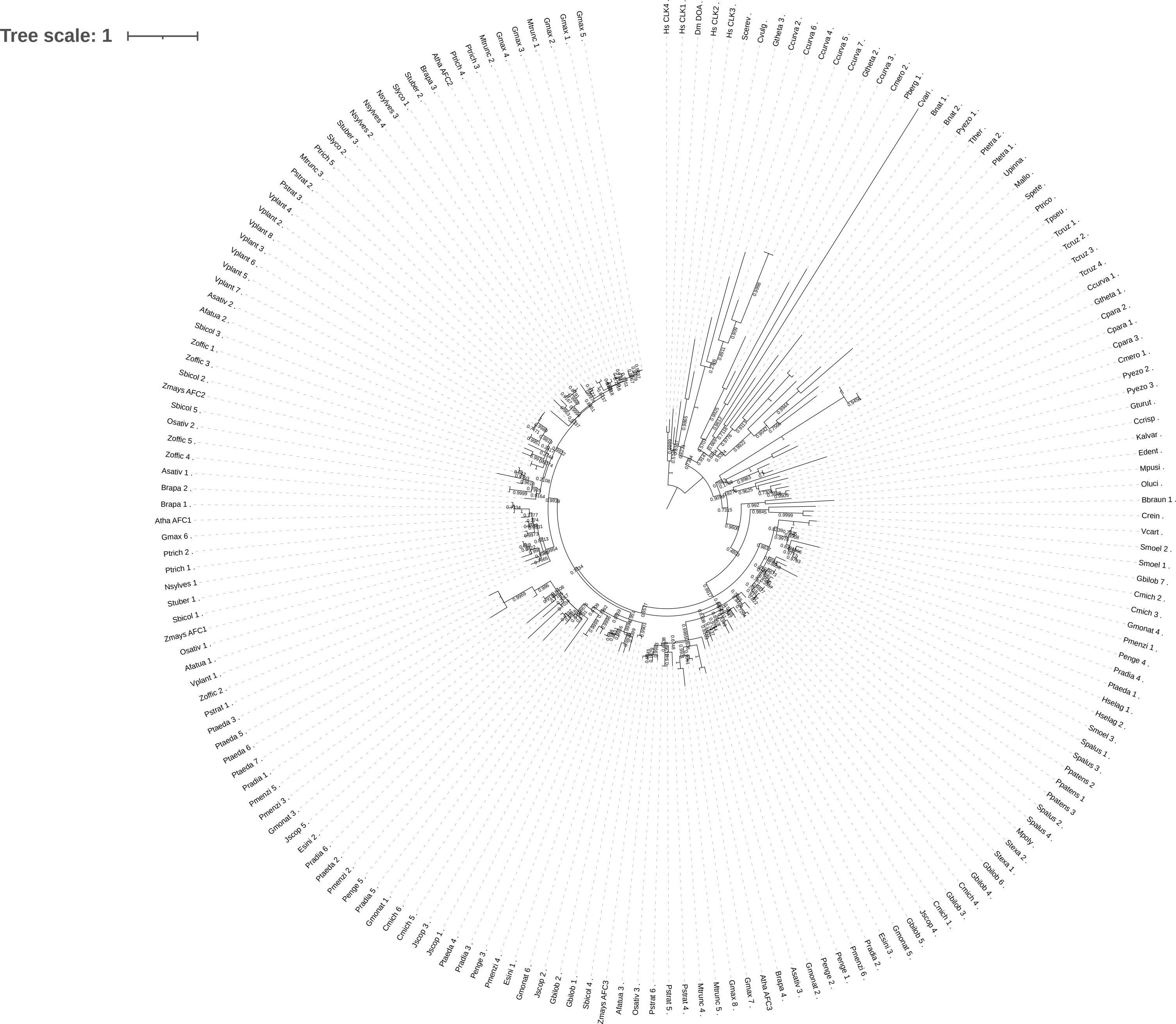
Tree scale: 1
Hs CLK4 .
Hs CLK1 .
Dm DOA .
Hs CLK2 .
Hs CLK3 .
Scerev .
Cvulg .
Gtheta 3 .
Ccurva 2 .
Ccurva 6 .
Ccurva 4 .
Ccurva 5 .
Ccurva 7 .
Gtheta 2 .
Ccurva 3 .
Cmero 2 .
Pberg 1 .
Cvari .
Bnat 1 .
Bnat 2 .
Pyezo 1 .
Tther .
Ptetra 2 .
Ptetra 1 .
Upinna .
Mallo .
Spete .
Ptrico .
Tpseu .
Tcruz 1 .
Tcruz 2 .
Tcruz 3 .
Tcruz 4 .
Ccurva 1 .
Gtheta 1 .
Cpara 2 .
Cpara 1 .
Cpara 3 .
Cmero 1 .
Pyezo 2 .
Pyezo 3 .
Gturut .
Ccrisp .
Kalvar .
Edent .
Mpusi .
Oluci .
Bbraun 1 .
Crein .
Vcart .
Smoel 2 .
Smoel 1 .
Gbilob 7 .
Cmich 2 .
Cmich 3 .
Gmonat 4 .
Pmenzi 1 .
Penge 4 .
Pradia 4 .
Ptaeda 1 .
Hselag 1 .
Hselag 2 .
Smoel 3 .
Spalus 1 .
Spalus 3 .
Ppatens 2
Ppatens 1 .
Ppatens 3
Spalus 2 .
Spalus 4 .
Mpoly .
Stexa 2 .
Stexa 1 .
Gbilob 6 .
Gbilob 4 .
Cmich 4 .
Gbilob 3 .
Cmich 1 .
Jscop 4 .
Gbilob 5 .
Gmonat 5 .
Esini 3 .
Pradia 2 .
Pmenzi 6 .
Penge 1 .
Penge 2 .
Gmonat 2 .
Asativ 3 .
Brapa 4 .
Atha AFC3
Gmax 7 .
Gmax 8 .
Mtrunc 5 .
Mtrunc 4 .
Pstrat 4 .
Pstrat 5 .
Pstrat 6 .
Osativ 3 .
Afatua 3 .
Zmays AFC3
Sbicol 4 .
Gbilob 1 .
Gbilob 2 .
Jscop 2 .
Gmonat 6 .
Esini 1 .
Pmenzi 4 .
Penge 3 .
Pradia 3 .
Ptaeda 4 .
Jscop 1 .
Jscop 3 .
Cmich 5 .
Cmich 6 .
Gmonat 1 .
Pradia 5 .
Penge 5 .
Pmenzi 2 .
Ptaeda 2 .
Pradia 6 .
Esini 2 .
Jscop 5 .
Gmonat 3 .
Pmenzi 3 .
Pmenzi 5 .
Pradia 1 .
Ptaeda 7 .
Ptaeda 6 .
Ptaeda 5 .
Ptaeda 3 .
Pstrat 1 .
Zoffic 2 .
Vplant 1 .
Afatua 1 .
Osativ 1 .
Zmays AFC1
Sbicol 1 .
Stuber 1 .
Nsylves 1 .
Ptrich 1 .
Ptrich 2 .
Gmax 6 .
Atha AFC1
Brapa 1 .
Brapa 2 .
Asativ 1 .
Zoffic 4 .
Zoffic 5 .
Osativ 2 .
Sbicol 5 .
Zmays AFC2
Sbicol 2 .
Zoffic 3 .
Zoffic 1 .
Sbicol 3 .
Afatua 2 .
Asativ 2 .
Vplant 7 .
Vplant 5 .
Vplant 6 .
Vplant 3 .
Vplant 8 .
Vplant 2 .
Vplant 4 .
Pstrat 3 .
Pstrat 2 .
Mtrunc 3 .
Ptrich 5 .
Slyco 2 .
Stuber 3 .
Nsylves 2
Nsylves 4
Nsylves 3
Slyco 1 .
Stuber 2 .
Brapa 3 .
Atha AFC2
Ptrich 4 .
Ptrich 3 .
Mtrunc 2 .
Gmax 4 .
Gmax 3 .
Mtrunc 1 .
Gmax 2 .
Gmax 1 .
Gmax 5 .

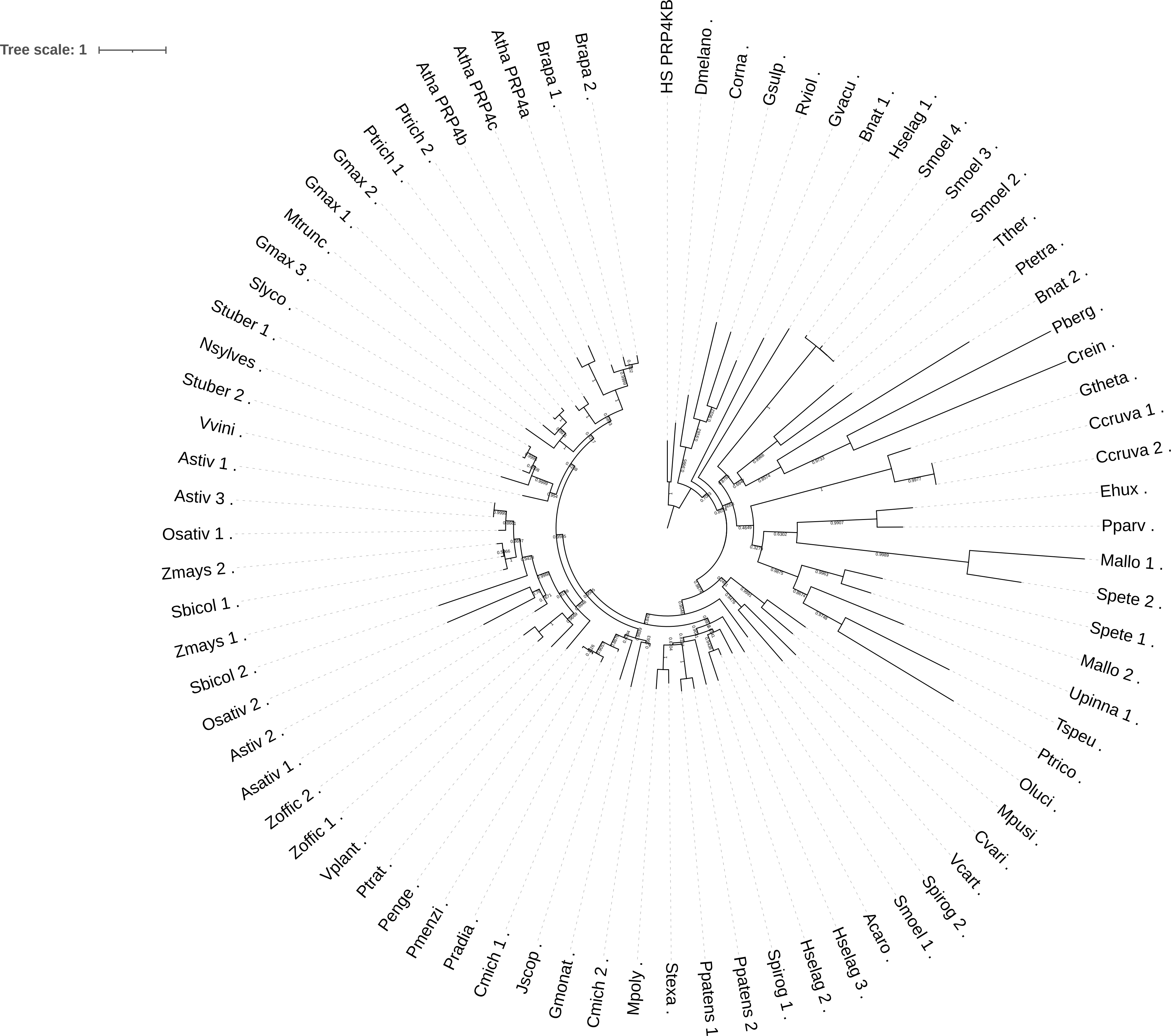
Tree scale: 1
HS PRP4KB
Dmelano .
Corna .
Gsulp .
Rviol .
Gvacu .
Bnat 1 .
Hselag 1 .
Smoel 4 .
Smoel 3 .
Smoel 2 .
Tther .
Ptetra .
Bnat 2 .
Pberg .
Crein .
Gtheta .
Ccruva 1 .
Ccruva 2 .
Ehux .
Pparv .
Mallo 1 .
Spete 2 .
Spete 1 .
Mallo 2 .
Upinna 1 .
Tspeu .
Ptrico .
Oluci .
Mpusi .
Cvari .
Vcart .
Spirog 2 .
Smoel 1 .
Acaro .
Hselag 3 .
Hselag 2 .
Spirog 1 .
Ppatens 2
Ppatens 1
Stexa .
Mpoly .
Cmich 2 .
Gmonat .
Jscop .
Cmich 1 .
Pradia .
Pmenzi .
Penge .
Ptrat .
Vplant .
Zoffic 1 .
Zoffic 2 .
Asativ 1 .
Astiv 2 .
Osativ 2 .
Sbicol 2 .
Zmays 1 .
Sbicol 1 .
Zmays 2 .
Osativ 1 .
Astiv 3 .
Astiv 1 .
Vvini .
Stuber 2 .
Nsylves .
Stuber 1 .
Slyco .
Gmax 3 .
Mtrunc .
Gmax 1 .
Gmax 2 .
Ptrich 1 .
Ptrich 2 .
Atha PRP4b
Atha PRP4c
Atha PRP4a
Brapa 1 .
Brapa 2 .
