# Supplementary figures and images for: Genome-scale analysis of Arabidopsis splicing-related protein kinase families reveals roles in abiotic stress adaptation
Source: BMC Plant Biol. 2022 Oct 22;22:496. doi: 10.1186/s12870-022-03870-9 (PMC9587599; doi:10.1186/s12870-022-03870-9)

## SHOOTS

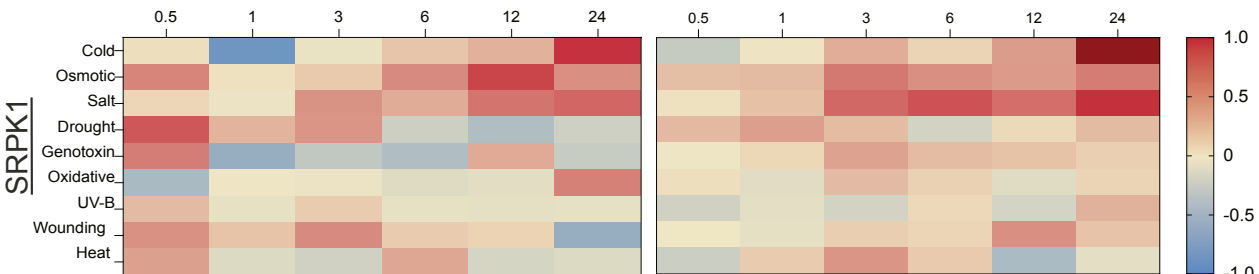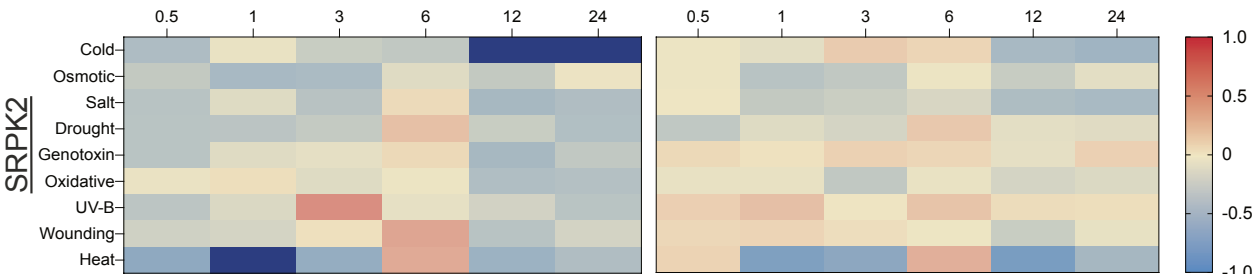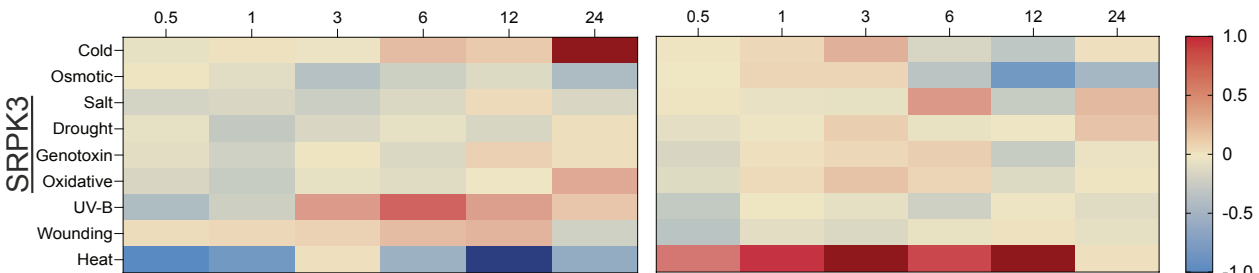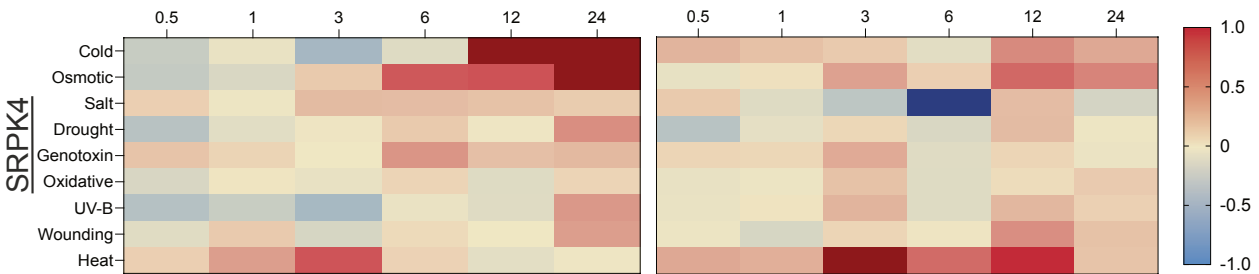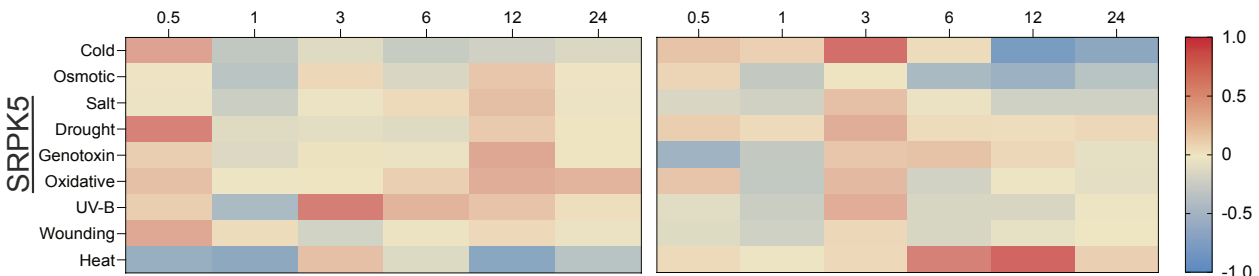

## ROOTS

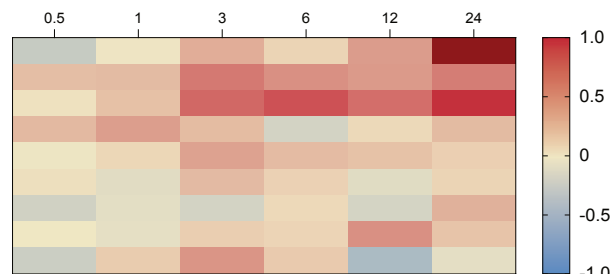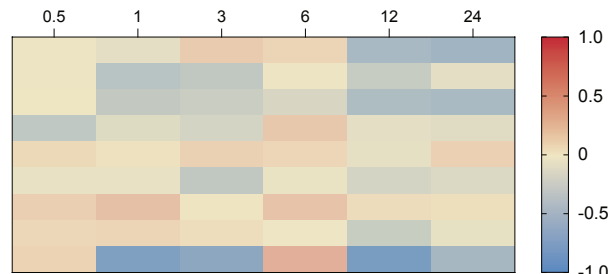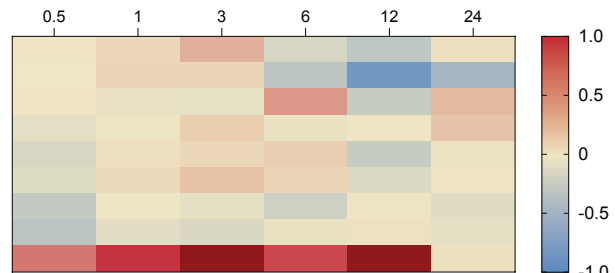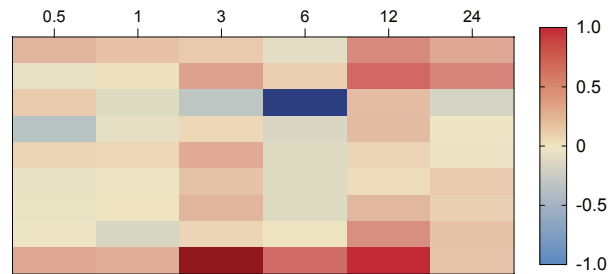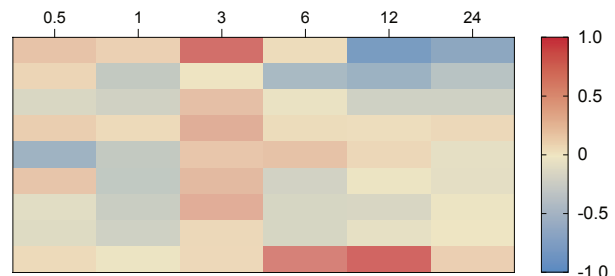

Supplement: Supplementary file 17 — Additional file 17. [file 12870_2022_3870_MOESM17_ESM.pdf]

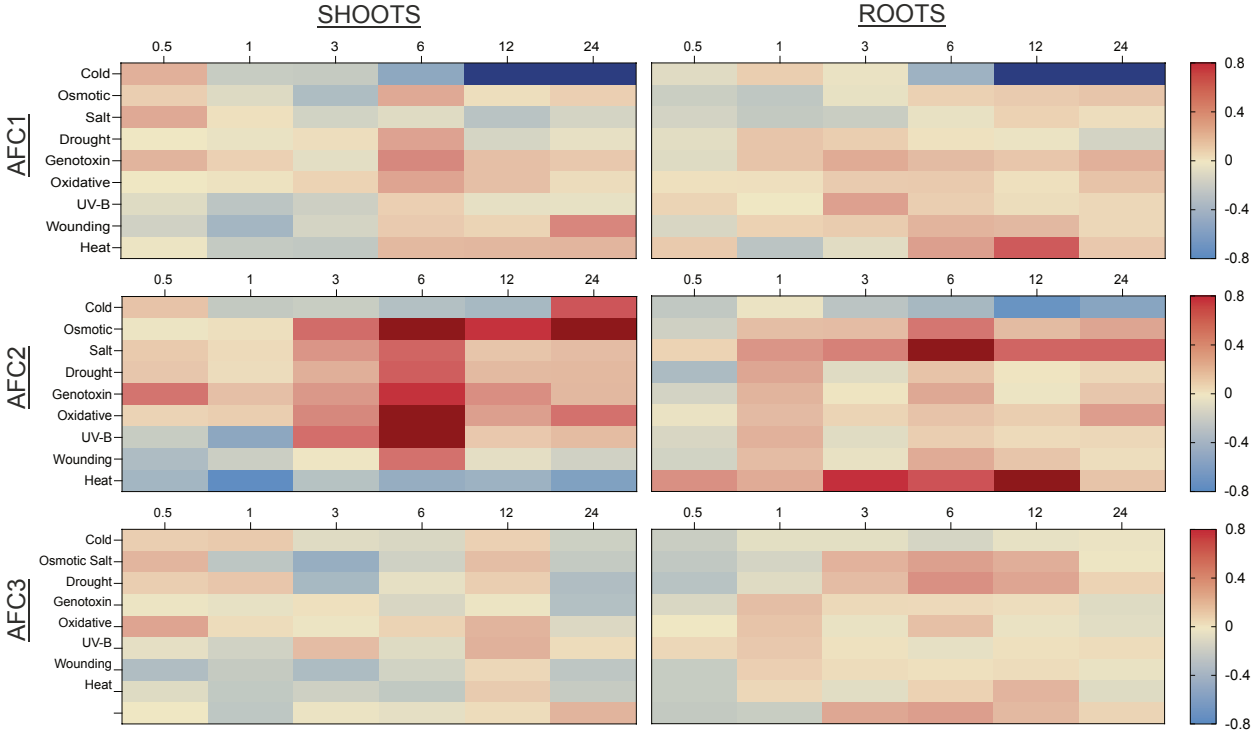

Supplement: Supplementary file 18 — Additional file 18. [file 12870_2022_3870_MOESM18_ESM.pdf]

PRP4KA

SHOOTS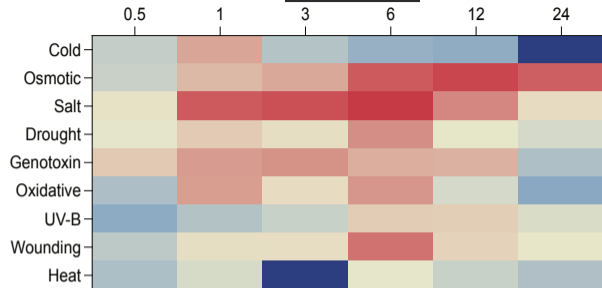ROOTS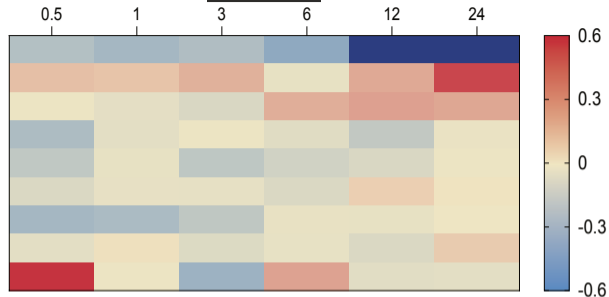

PRP4KB

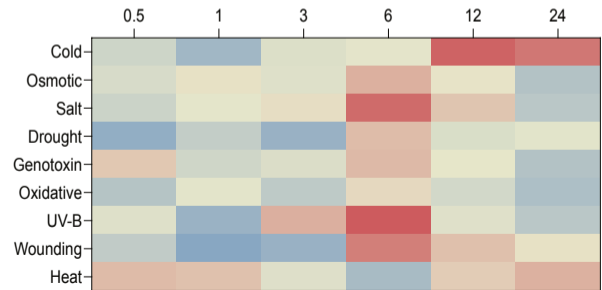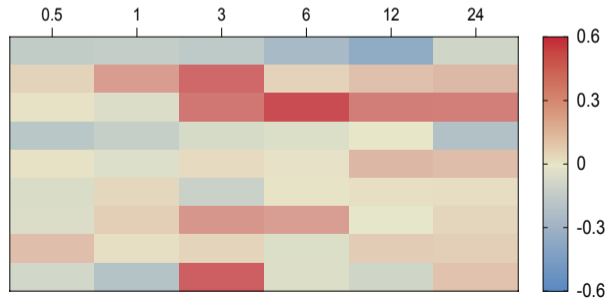

Supplement: Supplementary file 19 — Additional file 19. [file 12870_2022_3870_MOESM19_ESM.pdf]

SRPKs 24:0

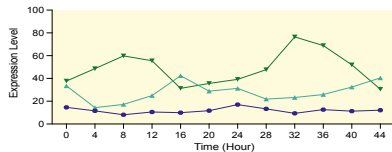

SRPKs 12:12

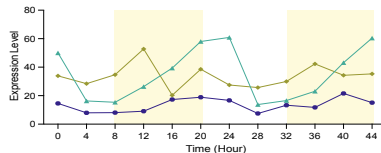

SRPKs 8:16

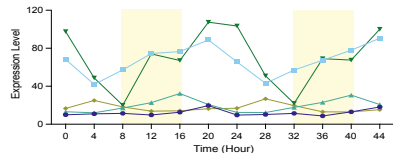

AFCs 24:0

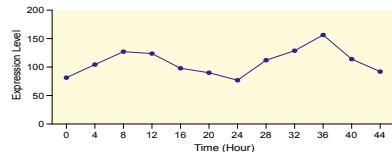

AFCs 12:12

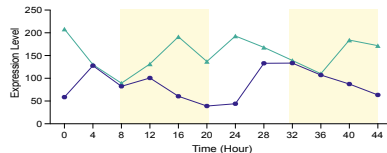

AFCs 8:16

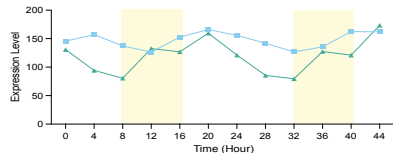

PRP4Ks 24:0

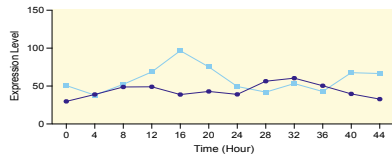

PRP4Ks 12:12

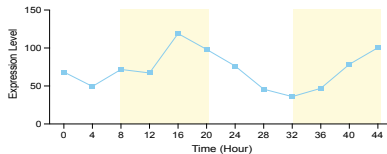

PRP4Ks 8:16

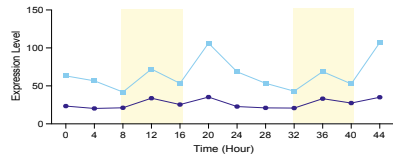

Supplement: Supplementary file 20 — Additional file 20. [file 12870_2022_3870_MOESM20_ESM.pdf]

**A**

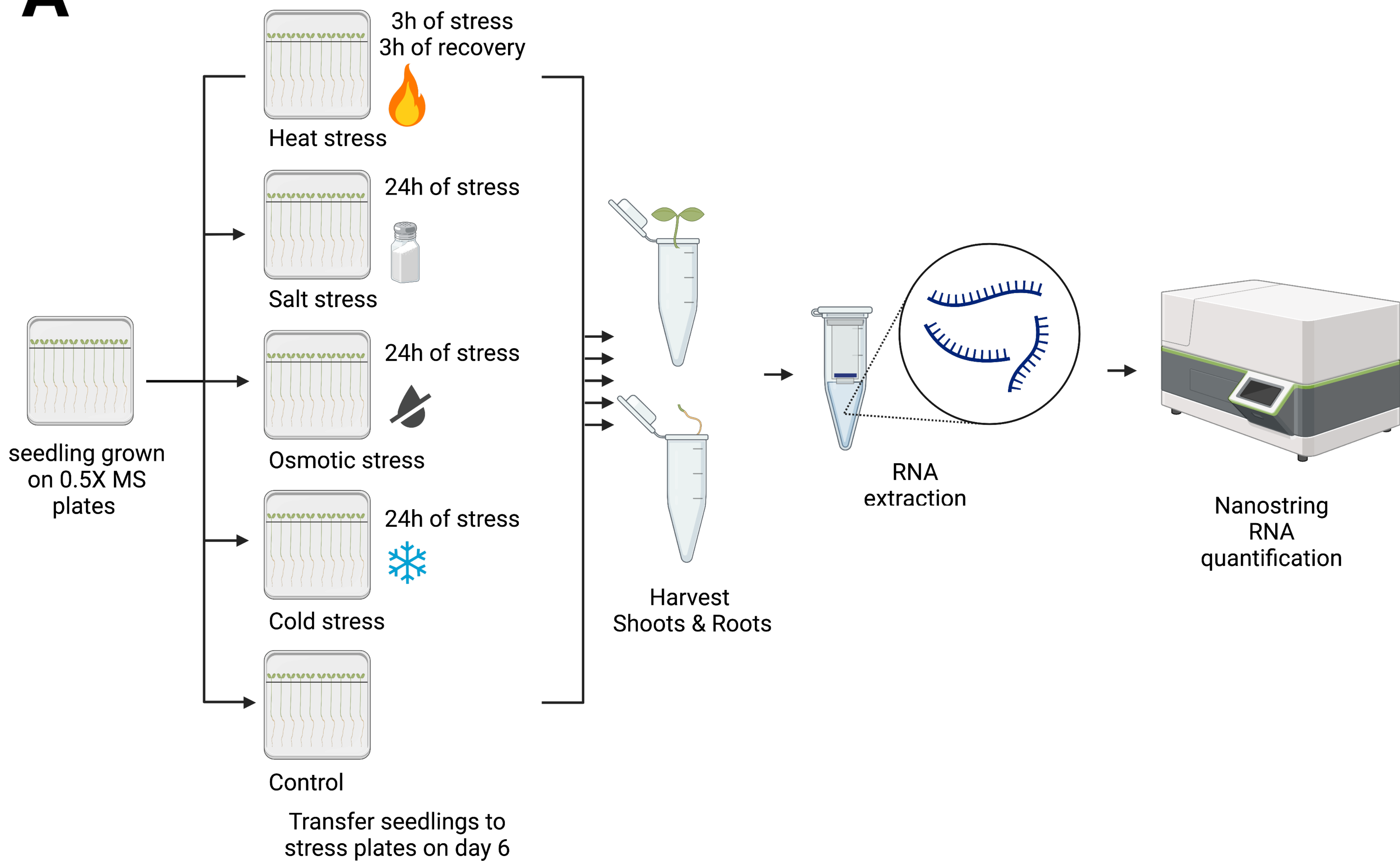

**B**

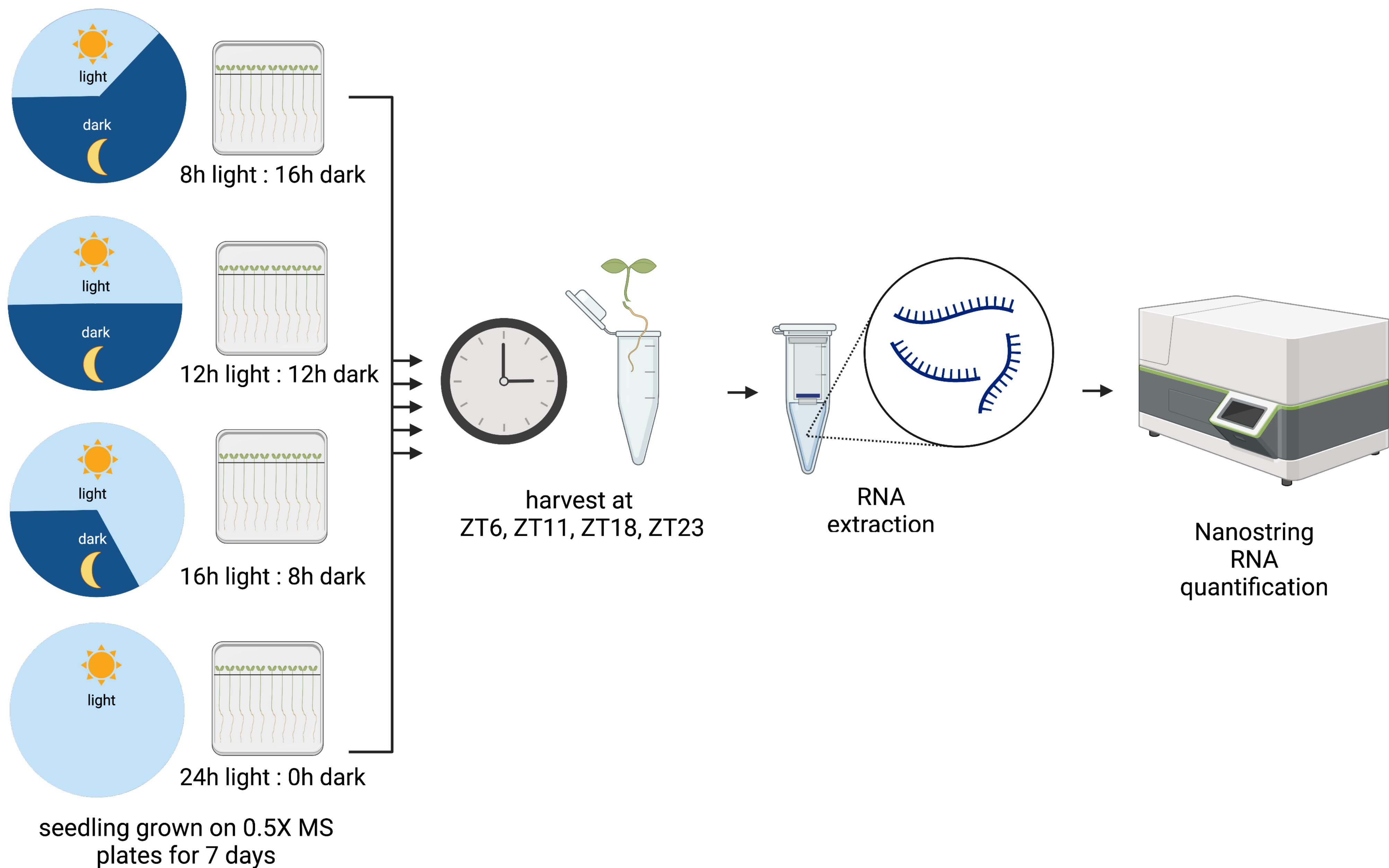

Supplement: Supplementary file 21 — Additional file 21. [file 12870_2022_3870_MOESM21_ESM.pdf]

## SHOOTS

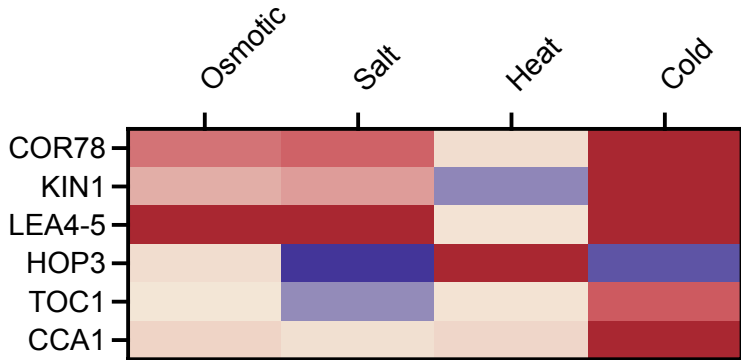

## ROOTS

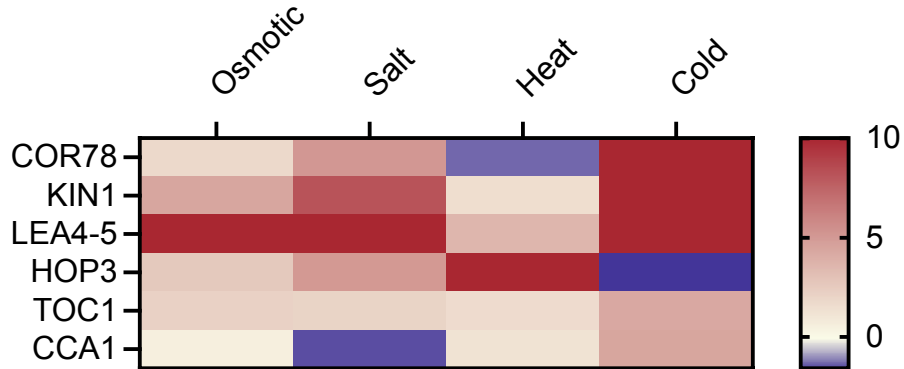

Supplement: Supplementary file 22 — Additional file 22. [file 12870_2022_3870_MOESM22_ESM.pdf]

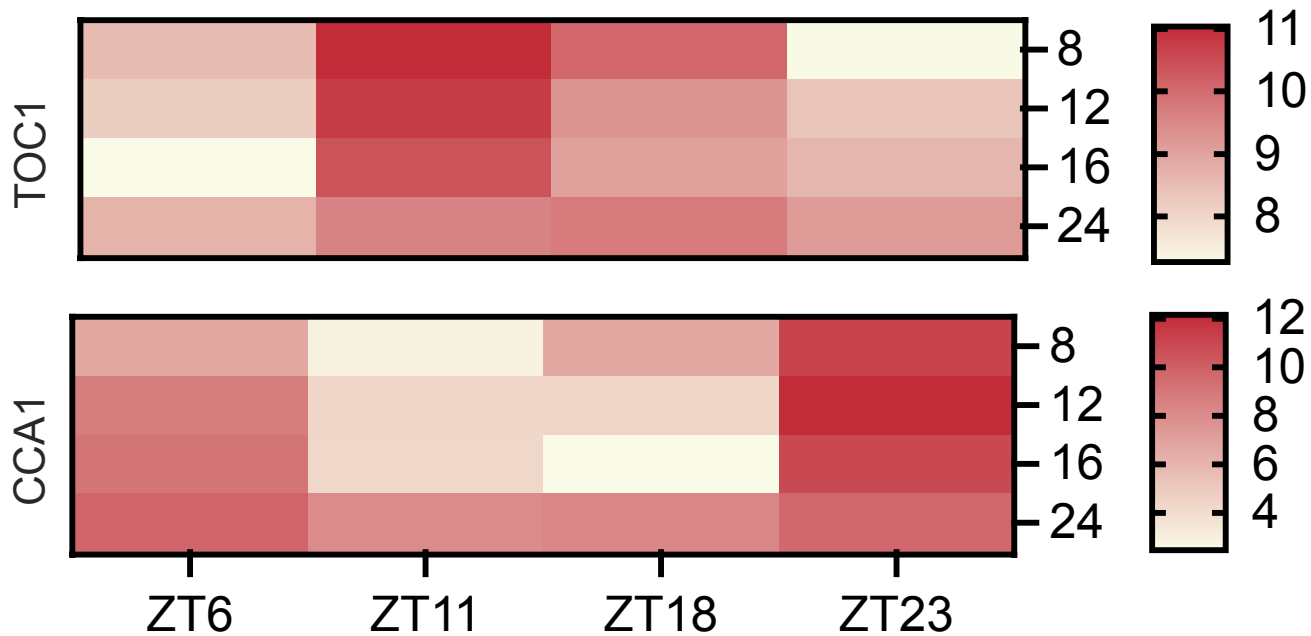

Supplement: Supplementary file 23 — Additional file 23. [file 12870_2022_3870_MOESM23_ESM.pdf]
