# Supplementary material for: Genome-scale analysis of Arabidopsis splicing-related protein kinase families reveals roles in abiotic stress adaptation
Source: BMC Plant Biol. 2022 Oct 22;22:496. doi: 10.1186/s12870-022-03870-9 (PMC9587599; doi:10.1186/s12870-022-03870-9)
Supplement: Supplementary file 16 — Additional file 16. [file 12870_2022_3870_MOESM16_ESM.pdf]

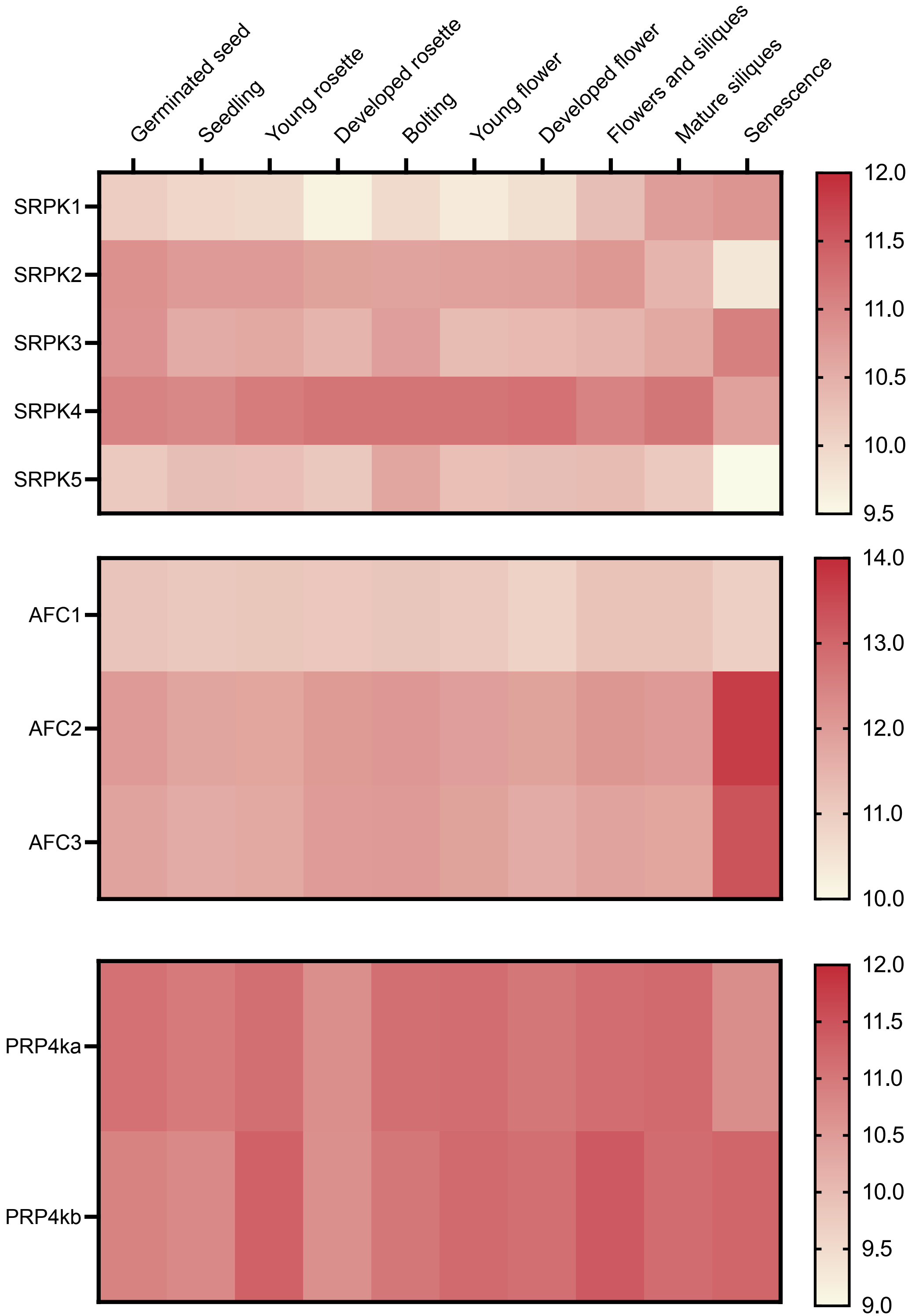
Germinated seed
Seedling
Young rosette
Developed rosette
Bolting
Young flower
Developed flower
Flowers and siliques
Mature siliques
Senescence
SRPK1
SRPK2
SRPK3
SRPK4
SRPK5
12.0
11.5
11.0
10.5
10.0
9.5
AFC1
AFC2
AFC3
14.0
13.0
12.0
11.0
10.0
PRP4ka
PRP4kb
12.0
11.5
11.0
10.5
10.0
9.5
9.0
